# Supplementary material for: The Covalent Linking of Organophosphorus Heterocycles to Date Palm Wood-Derived Lignin: Hunting for New Materials with Flame-Retardant Potential
Source: Molecules. 2023 Dec 1;28(23):7885. doi: 10.3390/molecules28237885 (PMC10707890; doi:10.3390/molecules28237885)
Supplement: Supplementary file 1 [file molecules-28-07885-s001.zip › molecules-2688741-supplementary.pdf]

Supplementary Information

The Covalent Linking of Organophosphorus Heterocycles to Date Palm Wood-Derived Lignin: Hunting for New Materials with Flame-Retardant Potential

Contents

General Considerations	S2
Lignin General Procedures	S3-S4
Figure S1	S5
Figure S2	S5
Figure S3	S5
Table S1	S6
Table S2	S6
Figure S4	S7
Figure S5	S8
Figure S6	S8
Figure S7	S9
Lignin Model Compound Synthesis	S10-S11
Table S3	S12
Table S4	S12
NMR Spectra of compounds and lignins	S13-S36
Supplementary Material References	S37

General Considerations

Commercially available compounds were purchased and used as received unless otherwise stated. Purified tosyl chloride was prepared as follows: commercially available tosyl chloride was dissolved in chloroform (2.5 mL/g) and diluted with hexane (12.5 mL/g). Activated charcoal was added and after stirring, the suspension was filtered through a Celite pad. The filtrate was concentrated under vacuum and dried under vacuum at 50 °C for 24 hours to afford purified tosyl chloride.

Solution-state NMR spectra were obtained using a Bruker AV-III 500 fitted with CryoProbe Prodigy BBO and a Bruker AV-III-HD 700 fitted with CryProbe Prodigy TCI. NMR data were processed using MestReNova v12.0.3. Further processing of spectra for figures was carried out using Adobe Illustrator 2023 v27.8.

IR spectra were obtained using a Shimadzu IRAffinity 1S IR Spectrometer as ATR. The IR data were processed using OriginPro 2022 v9.9.0.225.

Mass spectrometry data were acquired through the University of St Andrews School of Chemistry mass spectrometry service.

TGA data were obtained using a Netzsch STA 449C. Approximately 15 mg of sample was weighed into an alumina crucible for each measurement. Measurements were performed under nitrogen flow (50 mL/min) at a heating rate of 10 °C/min. TGA data were processed using OriginPro 2022 v9.9.0.225.

Lignin General Procedures

Butanosolv Extraction

Date palm wood (DPW) biomass was pretreated using an optimised butanosolv pretreatment as previously reported.[1] In brief, DPW biomass (19.7 g, dried to constant weight) was suspended in ethanol (200 mL) and stirred at rt for 16 h. The suspension was filtered and the residue re-suspended in fresh ethanol (200 mL), stired for a further 4 h, filtered and dried under vacuum at 50 °C for 16 h. The ethanol extracts were combined and concentrated to afford an ethanol soluble extractives component (0.67 g, 3 wt%). The washed DPW biomass was then suspended in water (500 mL), heated at reflux for 4 h, filtered and dried under vacuum at 50 °C for 16 h. The aqueous filtrate was precipitated into ice-cold ethanol (1 L) and collected as an ethanol insoluble component (0.72 g, 4 wt%) and an ethanol soluble component (1.76 g, 9 wt%). The remaining DPW biomass was then suspended in 19:1 *n*-butanol/4M HCl (200 mL) and heated at reflux for 6 h then filtered, rinsing the pulp with acetone (2 x 100 mL). The pulp was dried under vacuum at 50 °C for 16 h to afford a cellulose pulp component (7.78 g, 40 wt%). The filtrate was concentrated under reduced pressure, dissolved in the minimum volume of 9:1 acetone/water and precipitated into 10 v/v eq. ice-cold water. The filtrate was concentrated under reduced pressure, extracted with 1:1 acetone/methanol (200 mL), filtered to remove inorganic salts and concentrated under reduced pressure to afford a hemicellulose sugar component (6.59 g, 34 wt%). The residue was collected by filtration, air dried, re-dissolved in the minimum volume of 9:1 acetone/methanol and precipitated into 10 v/v eq. 1:1 hexane/ether. The solid was collected by filtration and dried under vacuum at 50 °C for 16 h to afford the date palm wood lignin (**DPW Lignin**) component (1.59 g, 8 wt%). The filtrate was concentrated under reduced pressure to afford and organic solvent soluble extractives component (1.34 g, 7 wt%). Total recovered mass was 20.45 g (105 wt%, The >100% mass balance likely results from: (i) challenges in drying the viscous hemicellulose sugar component and (ii) butanol incorporation into several of the resulting fractions.

Sodium Hydroxide Ester Hydrolysis

DPW Lignin (0.60 g) was suspended in 0.1M aqueous NaOH (20 mL/g, 12 mL), heated to 50 °C with stirring for 12 h, and then precipitated into 10 v/v eq. 0.1 HCl, filtered, dried under vacuum at 50 °C for 16 h to afford deacylated lignin (**DeAcyl Lignin**, 0.43 g, 72 wt%). The filtrate was concentrated under reduced pressure and a sample collected for NMR analysis.

Lignin Tosylation

Using a literature procedure,[2] lignin (1 wt eq.) and freshly purified tosyl chloride (2.5 wt eq.) were dissolved in pyridine (10 mL/g) and stirred at rt for 16 h. The modified lignin was precipitated into 10 v/v eq. 0.1M HCl, filtered under suction to dryness, redissolved in the minimum volume of acetone, precipitated into 10 v/v eq. ice-cold ethanol, filtered and dried under vacuum at 50 °C for 16 h. The crude tosylated lignin was then purified by column chromatography on silica gel, eluting with 0-100% DCM/hexane then 0-10% methanol/DCM then dried under vacuum at 50 °C for 16 h to afford tosylated lignin (**DPW Lignin Ts** or **DeAcyl Lignin Ts**).

Lignin Azidation

Using a literature procedure,[2] tosylated lignin (1 wt eq.) and sodium azide (2.5 wt eq.) were suspended in DMF (10 mL/g) and heated at 50 °C for 16 h. The modified lignin was precipitated into 10 v/v eq. 0.1M HCl, filtered and dried under vacuum at 50 °C for 16 h. The crude azidated lignin was then purified by column chromatography on silica gel, eluting with 0-100% DCM/hexane then 0-10%

methanol/DCM then dried under vacuum at 50 °C for 16 h to afford azidated lignin (**DPW Lignin N_3** or **DeAcyl Lignin N_3**).

Lignin CuAAC Click Reaction

Using a literature procedure,[2] azidated lignin (1 wt eq.), organophosphorus alkyne (0.5 wt eq.), sodium ascorbate (0.5 wt eq.) and $CuSO_4.5H_2O$ (0.03 wt eq.) were dissolved in 5:1 DMF/water (10 mL/g) and stirred at rt for 16 h. The modified lignin was precipitated into 10 v/v eq. 0.1M HCl, filtered and dried under vacuum at 50 °C for 16 h. The crude final lignin was then purified by column chromatography on silica gel, eluting with 0-100% DCM/hexane then 0-10% methanol/DCM and dried under vacuum at 50 °C for 16 h to afford organophosphorus flame-retardant (OPFR) grafted lignin.

Lignin NMR Sample Preparation

Lignin (60.0 ± 0.1 mg) was dissolved in DMSO-d_6 (700 µL) and sonicated for 10 minutes to ensure complete dissolution. Samples were analysed using ^{1}H, HSQC, DOSY and ^{31}P NMR (where appropriate).

Phosphitylation for Quantitative ^{31}P NMR Analysis

From a literature procedure,[3] Lignin (30.0 ± 0.1 mg) was dissolved in 1:1.6 $CDCl_3$/pyridine (500 µL), containing cyclohexanol (10 µL) as an internal standard, and sonicated for 10 minutes to ensure complete dissolution. 2-Chloro-4,4,5,5-tetramethyl-1,3,2-dioxaphospholane (50 µL) was added and the combined sample diluted with $CDCl_3$ (500 µL). The quantitative ^{31}P NMR experiment was run within 6 h of sample preparation. The internal standard integral was set to 1.00 and referenced to 145.15 ppm.[4]

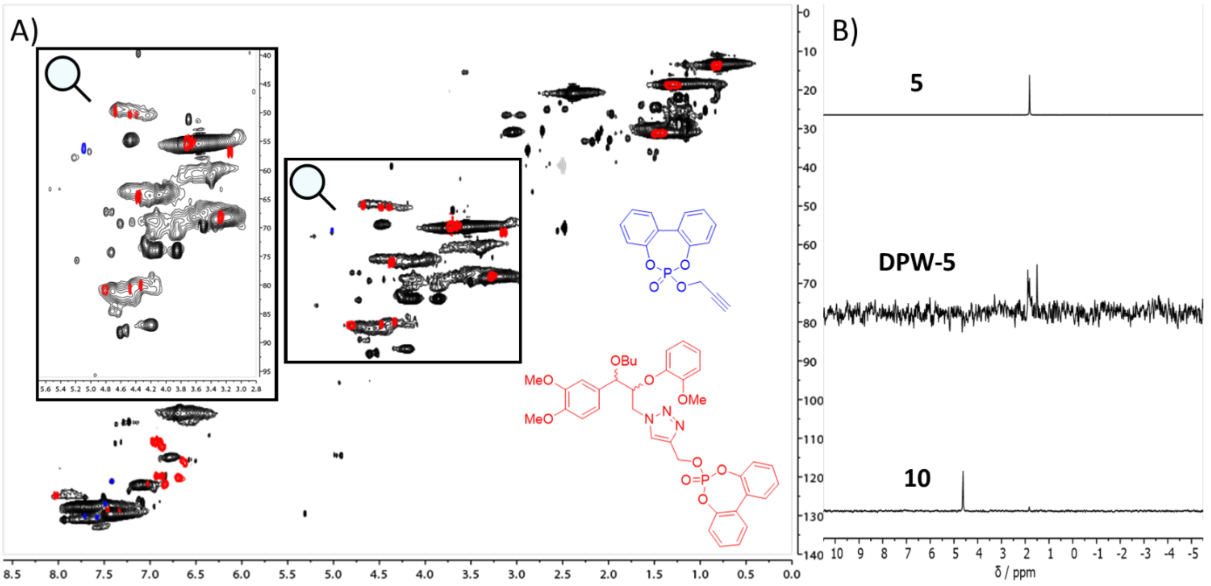

Figure S1. HSQC NMR (700 MHz, DMSO-d6) of **DPW Lignin** after attempted modification with **5** (to give **DPW-5**, black) overlaid with model compound **10** (red) and starting O-propargyl BPPO **5** (blue) with expansion of the linkage region. While overlap looks reasonably good in the linkage region, significant differences exist in the aromatic region.; B) ^{31}P NMR (202 MHz, DMSO-d6) spectra of the same samples. The signals in the HSQC that correspond to the CH2-azide functionality have disappeared, suggesting that an undesired side reaction took place leading to poor attachment. The ^{31}P NMR spectra supports a view that phosphorus environments similar to starting material **5** are present but that very little if any of a structure analogous to **10** has formed.

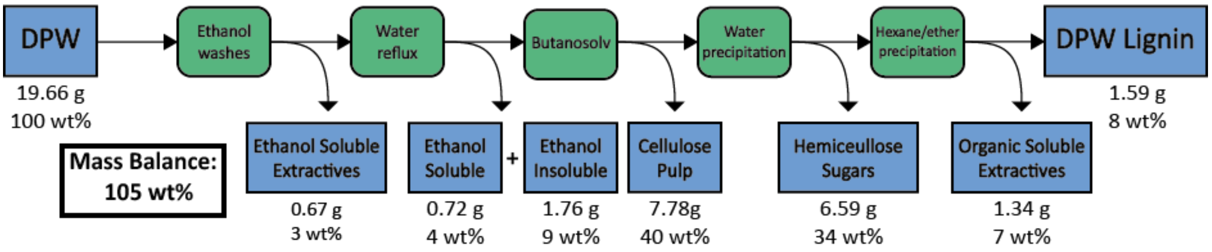

Figure S2. Mass balance of date palm wood biomass during pretreatment

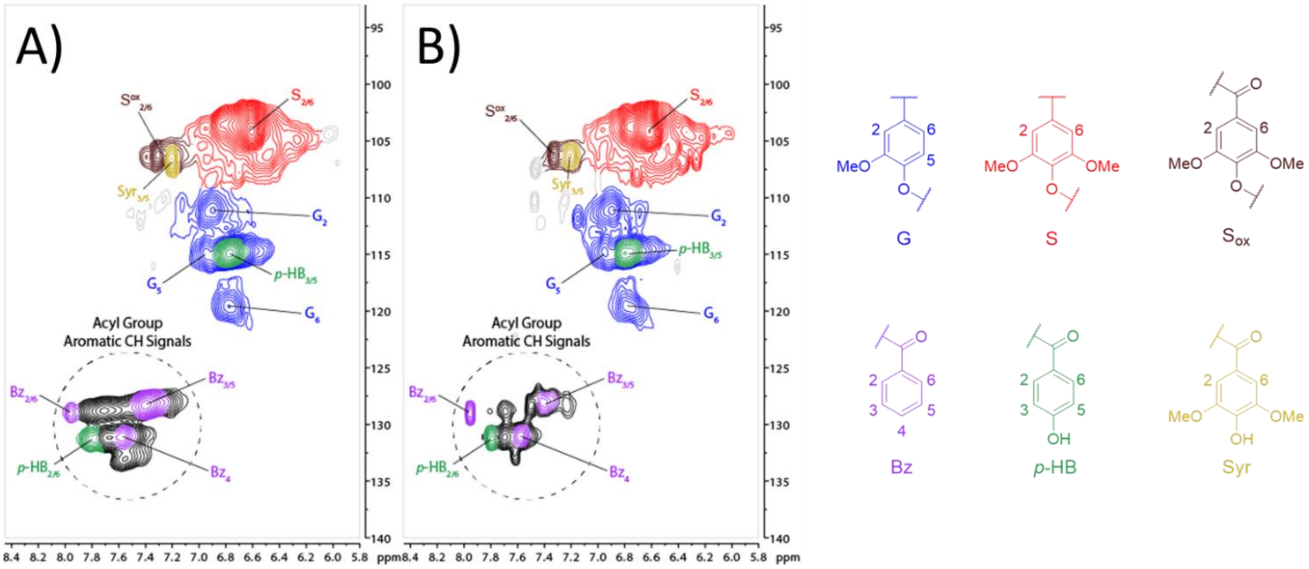

Figure S3. HSQC NMR (700 MHz, DMSO-d6) of the aromatic regions of A) **DPW Lignin** and B) **DeAcyl Lignin**

Table S1. Integrals of Quantitative ^{31}P NMR Relative to Internal Standard

Lignin	Sample Mass / mg	Relative Integral				
		Aliphatic	S	G	*p*-BH	COOH
DPW Lignin	30.1	0.61	0.22	0.08	0.13	0.00
DeAcyl Lignin	30.0	0.85	0.22	0.08	0.06	0.11

Table S2. Hydroxyl Content of Lignin Samples

Lignin	OH Content / mmol/g					
	Aliphatic	S	G	*p*-BH	COOH	Total Phenolic
DPW Lignin	6.8	2.4	0.9	1.4	0.0	4.8
DeAcyl Lignin	9.4	2.4	0.9	0.7	1.2	4.0

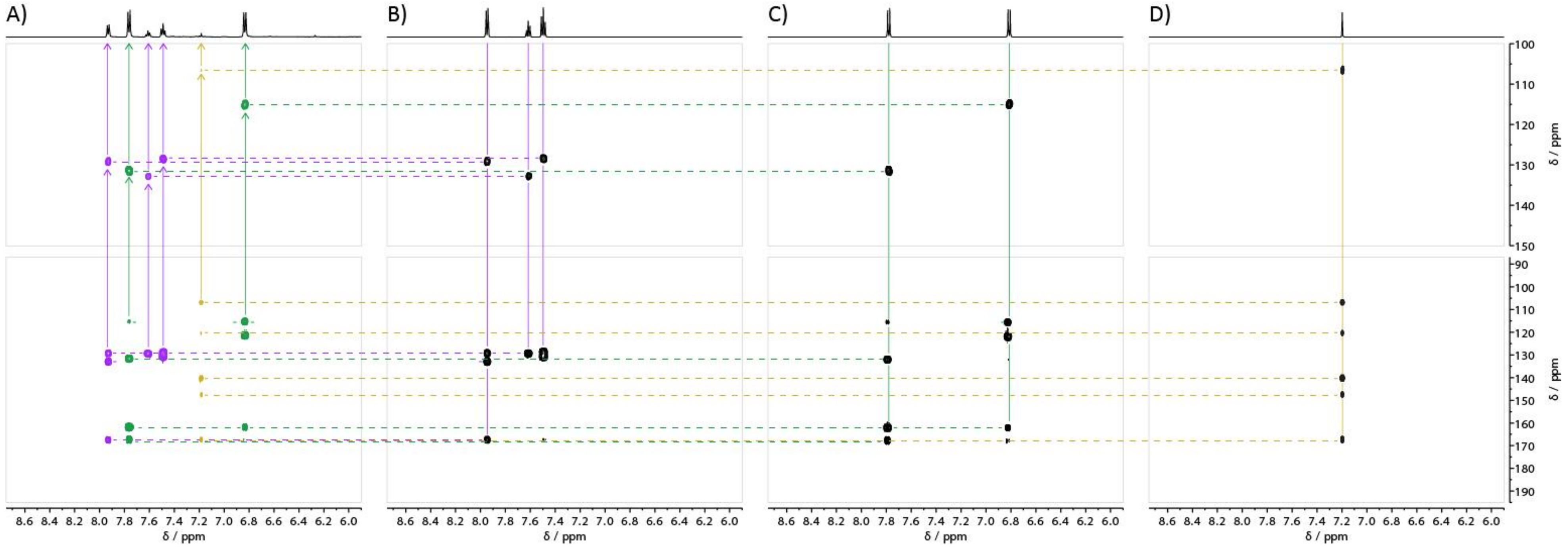

Figure S4. HSQC (top) and HMBC (bottom) NMR (500 MHz, DMSO-d$_6$) spectra of A) the recovered aqueous component following the sodium hydroxide deacylation treatment; B) benzoic acid; C) *p*-hydroxybenzoic acid; and D) syringic acid. The solid lines indicate the signal in the proton NMR spectra that correspond to the cross-peak signals in the 2D spectra. The dashed lines indicate signals that are equivalent between the 2D spectra. The clear correlation between signals is used to confirm the presence of benzoate, *p*-hydroxybenzoate, and syringic esters as pendant groups on the lignin chain and that these esters are converted to the corresponding carboxylic acids during the alkali hydrolysis, in agreement with literature.[5]

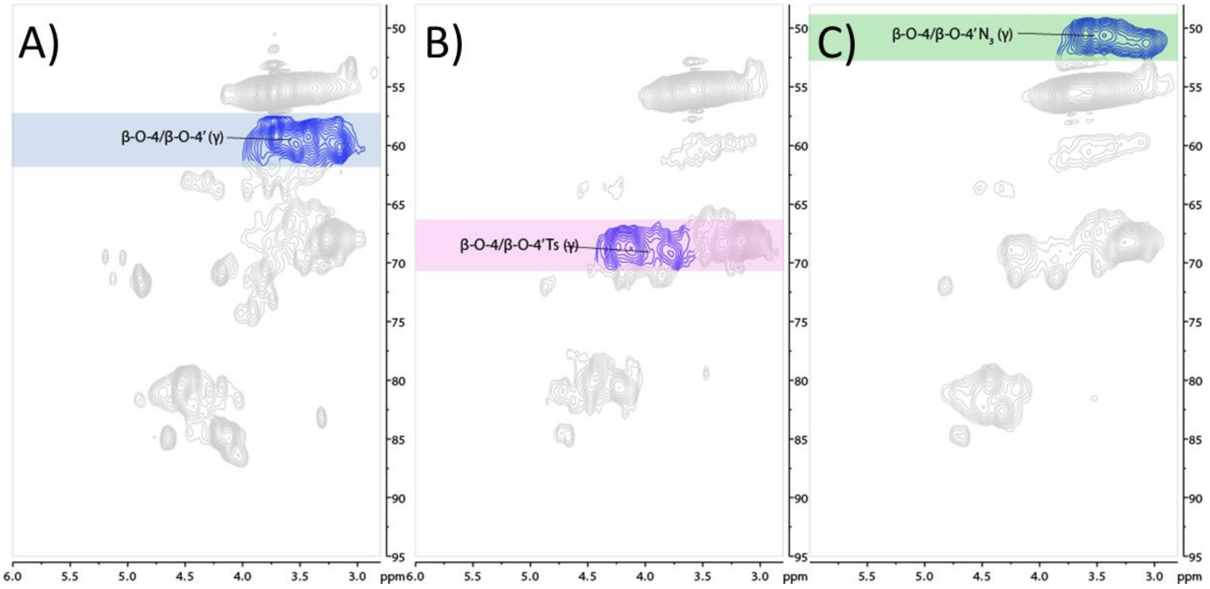

Figure S5. HSQC NMR (700 MHz, DMSO-d_6) spectra of A) deacylated date palm lignin (**DeAcyl Lignin**); B) deacylated tosylated date palm lignin (**DeAcyl Lignin Ts**); C) deacylated azidated date palm lignin (**DeAcyl Lignin-N$_3$**). Signals corresponding to the γ-position of the β-O-4 linking unit are highlighted in the spectra, showing the clear shift during each stage of the modification that confirms it has been successful.

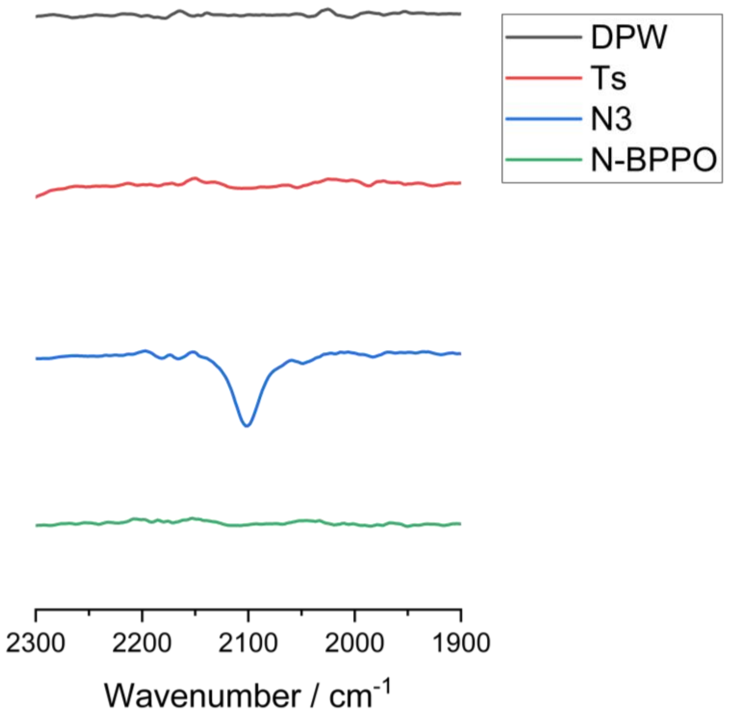

Figure S6. 2300 – 1900 cm^{-1} region of IR spectra of **DPW Lignin** (black), **DPW Lignin Ts** (red), **DPW Lignin N$_3$** and **DPW Lignin** modified with **6** (green). The signal at ~2100 cm^{-1} corresponds to the azide stretching frequency, with its appearance and disappearance through the reaction sequence confirming successful modification.

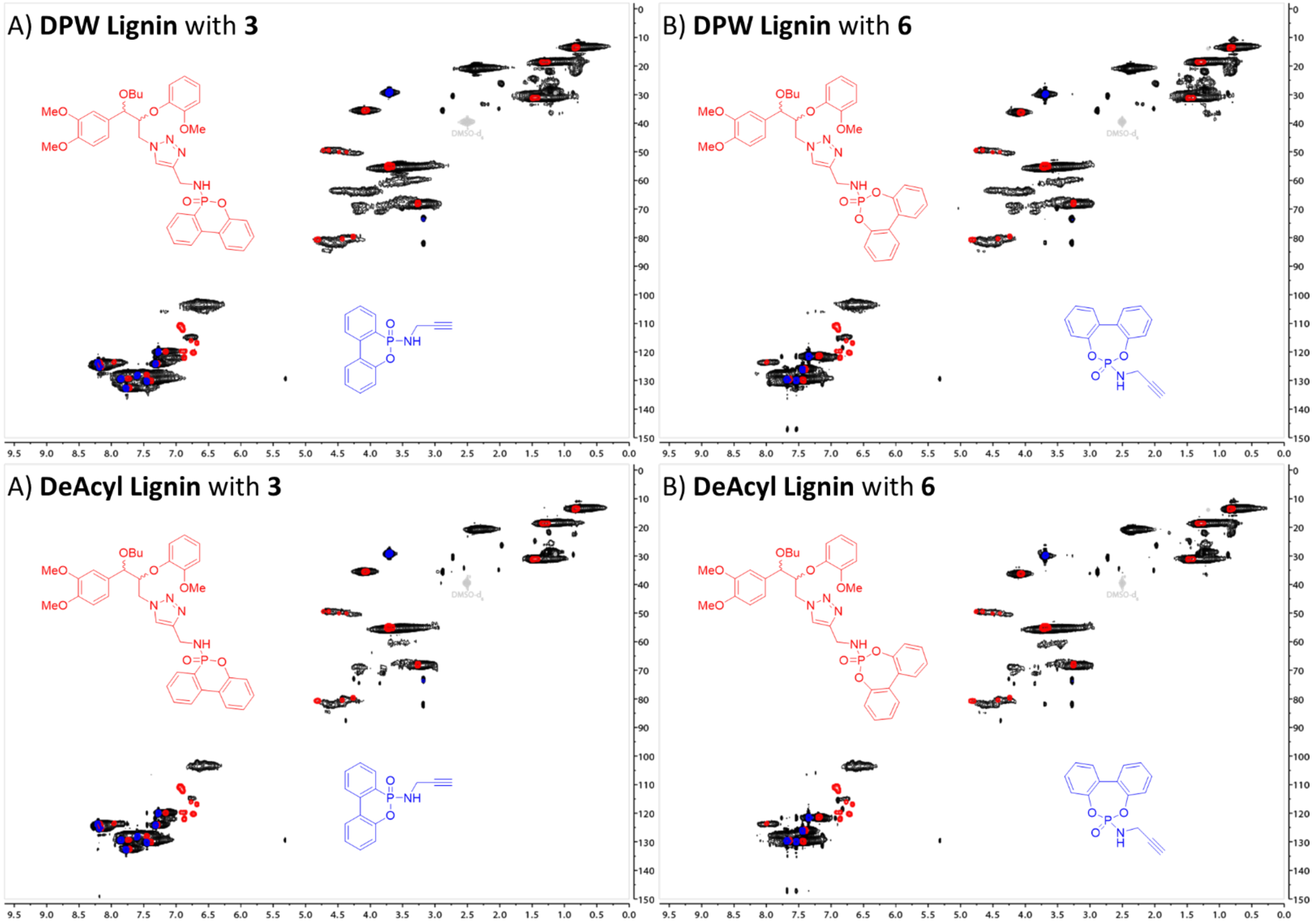

Figure S7. HSQC NMR (700 MHz for lignin, 500 MHz for small molecules, DMSO-d_6) of A) **DPW Lignin** grafted with **3** (black) overlaid with lignin dimer model compound **9** (red) and N-propargyl DOPO **3** (blue); B) **DPW Lignin** grafted with **6** (black) overlaid with lignin dimer model compound **11** (red) and N-propargyl BPPO **6** (blue); C) **DeAcyl Lignin** grafted with **3** (black) overlaid with lignin dimer model compound **9** (red) and N-propargyl DOPO **3** (blue); D) **DeAcyl Lignin** grafted with **6** (black) overlaid with lignin dimer model compound **11** (red) and N-propargyl BPPO **6** (blue). Overlay with both lignin dimer model compounds and small molecule OPFRs confirmed both attached and free OPFRs are present within the samples even after column chromatography.

Lignin Model Compound Synthesis General Procedure

Azide lignin model compound **8**[2] (1 eq., 50 mg), organophosphorus alkyne (1.1 eq.) sodium ascorbate (1.1 eq.) and copper sulfate pentahydrate (0.3 eq.) were dissolved in 5:1 DMF/water (2 mL) and stirred at rt for 16 h. The reaction was diluted with water (5 mL), extracted with DCM (3 x 5 mL), and the combined organic extracts washed with aq. sat. $NaHCO_3$ (10 mL), brine (10 mL), dried over anhydrous $MgSO_4$ and solvent removed under reduced pressure. The crude product was purified by column chromatography on silica gel eluting with EtOAc/hexane (0-95%) to afford clicked model compounds.

6-(((1-(3-butoxy-3-(3,4-dimethoxyphenyl)-2-(2-methoxyphenoxy)propyl)-1H-1,2,3-triazol-4-yl)methyl)amino)dibenzo[c,e][1,2]oxaphosphinine 6-oxide **9**

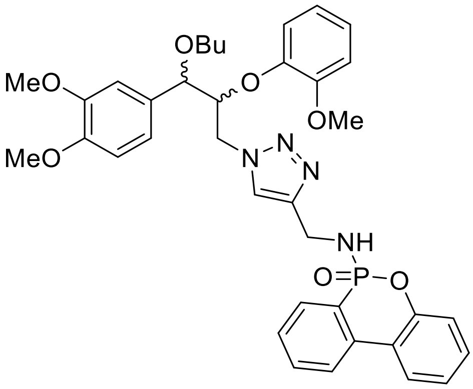

Prepared according to general procedure from azide lignin model compound **2** (50.2 mg, 0.12 mmol), **3** (36.2 mg, 0.13 mmol), sodium ascorbate (26.3 mg, 0.13 mmol) and copper sulfate pentahydrate (9.2 mg, 0.04 mmol). Afforded 6-(((1-(3-butoxy-3-(3,4-dimethoxyphenyl)-2-(2-methoxyphenoxy)propyl)-1H-1,2,3-triazol-4-yl)methyl)amino)dibenzo[c,e][1,2]oxaphosphinine 6-oxide **9** (71.9 mg, 85%) as a clear viscous oil that was a complex mixture of diastereomers. **HRMS** (ESI) calculated for $C_{37}H_{41}O_7N_4PNa$ $[M+Na]^+$ 707.2611; found 707.2587.

6-((1-(3-butoxy-3-(3,4-dimethoxyphenyl)-2-(2-methoxyphenoxy)propyl)-1H-1,2,3-triazol-4-yl)methoxy)dibenzo[d,f][1,3,2]dioxaphosphepine 6-oxide **10**

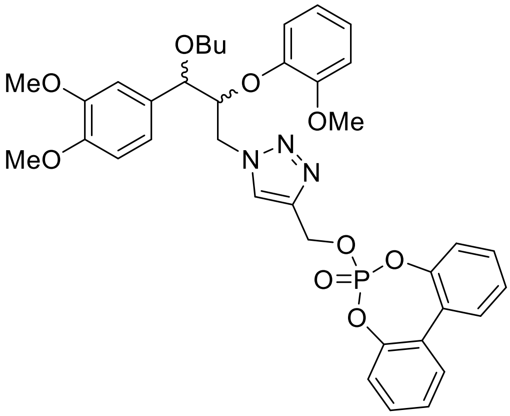

Prepared according to general procedure from azide lignin model compound **2** (49.5 mg, 0.12 mmol), **5** (38.2 mg, 0.13 mmol), sodium ascorbate (26.3 mg, 0.13 mmol) and copper sulfate pentahydrate (9.7 mg, 0.04 mmol). Afforded 6-((1-(3-butoxy-3-(3,4-dimethoxyphenyl)-2-(2-methoxyphenoxy)propyl)-1H-1,2,3-triazol-4-yl)methoxy)dibenzo[d,f][1,3,2]dioxaphosphepine 6-oxide **10** (21.1 mg, 25%) as a

colourless viscous oil that was a complex mixture of diastereomers. **HRMS** (ESI) calculated for $C_{37}H_{40}O_9N_3PNa$ $[M+Na]^+$ 724.2400; found 724.2407.

6-(((1-(3-butoxy-3-(3,4-dimethoxyphenyl)-2-(2-methoxyphenoxy)propyl)-1H-1,2,3-triazol-4-yl)methyl)amino)dibenzo[d,f][1,3,2]dioxaphosphepine 6-oxide **11**

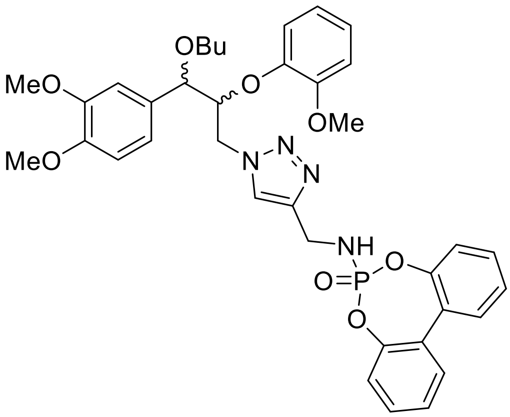

Prepared according to general procedure from azide lignin model compound **2** (49.7 mg, 0.12 mmol), **6** (39.8 mg, 0.14 mmol), sodium ascorbate (28.4 mg, 0.14 mmol) and copper sulfate pentahydrate (8.4 mg, 0.03 mmol). Afforded 6-(((1-(3-butoxy-3-(3,4-dimethoxyphenyl)-2-(2-methoxyphenoxy)propyl)-1H-1,2,3-triazol-4-yl)methyl)amino)dibenzo[d,f][1,3,2]dioxaphosphepine 6-oxide **11** (72.5 mg, 86%) as a colourless viscous oil that was a complex mixture of diastereomers. **HRMS** (ESI) calculated for $C_{37}H_{41}O_8N_4PNa$ $[M+Na]^+$ 723.2560; found 723.2539.

Table S3. Selected crystallographic data.

	3	5	6
formula	$C_{15}H_{12}NO_2P$	$C_{15}H_{11}O_4P$	$C_{15}H_{12}NO_3P$
fw	269.23	286.21	285.23
crystal description	Colourless plate	Orange prism	Colourless prism
crystal size [mm^3]	0.14 × 0.1 × 0.02	0.28 × 0.24 × 0.18	0.3 × 0.24 × 0.15
space group	$P2_1/c$	$Pca2_1$	$P\bar{1}$
a [Å]	8.8366(3)	12.3463(6)	8.25512(17)
b [Å]	17.6144(6)	8.5676(4)	8.5709(2)
c [Å]	8.8005(3)	25.4990(11)	10.4509(3)
α [°]			106.901(2)
β [°]	103.447(4)		92.495(2)
γ [°]			104.2120(19)
vol [Å]^3	1332.26(9)	2697.2(2)	680.56(3)
Z	4	8	2
ρ (calc) [g/cm^3]	1.342	1.410	1.392
μ [mm^{-1}]	1.807	0.213	0.208
F(000)	560.0	1184.0	296.0
reflections collected	18159	34166	14899
independent reflections (R_{int})	2504 (0.0659)	9464 (0.0839)	3218 (0.0252)
parameters, restraints	175, 1	361, 1	184, 1
GoF on F^2	1.239	1.057	1.092
R_1 [$I > 2\sigma(I)$]s	0.0582	0.0786	0.0300
wR_2 (all data)	0.1574	0.1518	0.0841
largest diff. peak/hole [e/Å^3]	0.42/-0.49	0.25/-0.37	0.33/-0.46

Table S4. Hydrogen-bond geometry lengths (Å) and angles (°).

	D–H	H···A	D···A	D–H···A
**3**				
N2–H2···O1^{i}	0.964(19)	1.88(2)	2.846(4)	176(4)
C23–H23···O1^{ii}	0.95	2.278(3)	3.208(6)	166.0(3)
**5**				
C21–H21B···O1^{iii}	0.99	2.377(4)	3.362(10)	173.6(6)
C51–H51A···O21^{vi}	0.99	2.378(4)	3.353(9)	168.2(7)
**6**				
N2–H2···O1^{v}	0.897(14)	2.017(14)	2.9101(13)	173.1(17)
C23–H23···O1^{vi}	0.95	2.2846(8)	3.2049(16)	162.92(10)

Symmetry Codes: (i) x-½, -y, z; (ii) x+½, -y, z; (iii) x+1, y, z; (vi) -x+1, -y+1, -z+2 (v) -x, -y, -z; (vi) x, y+1, z

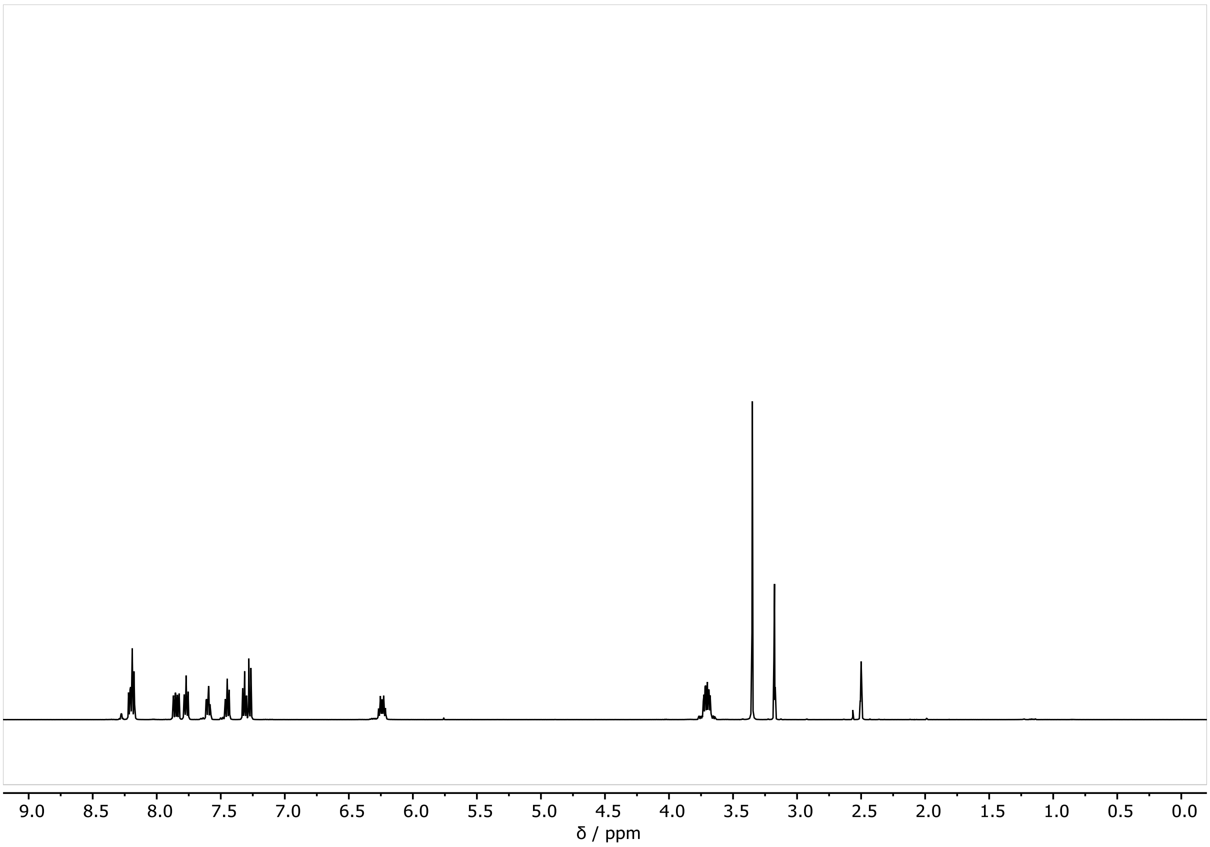

Figure S8. ^{1}H NMR (500 MHz, DMSO-d$_6$) of **3**

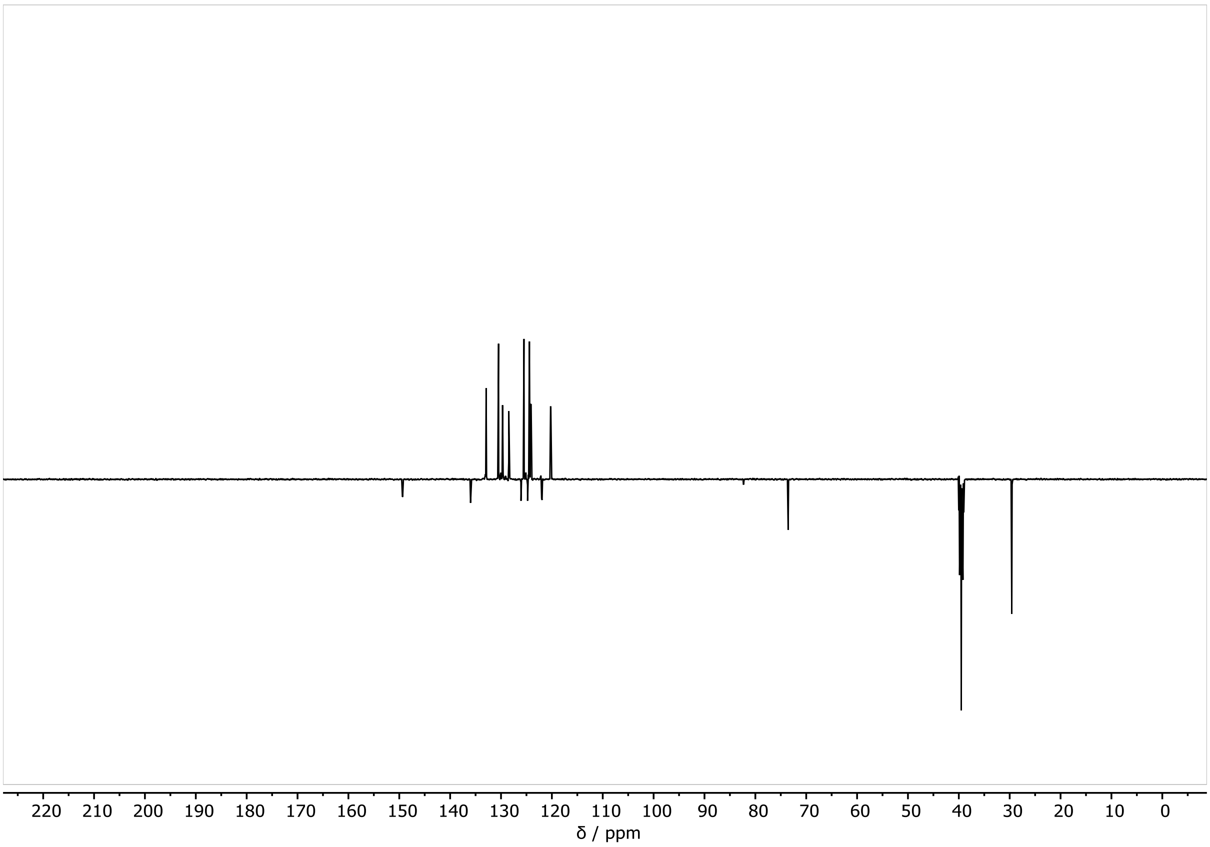

Figure S9. ^{13}C NMR (126 MHz, DMSO-d$_6$) of **3**

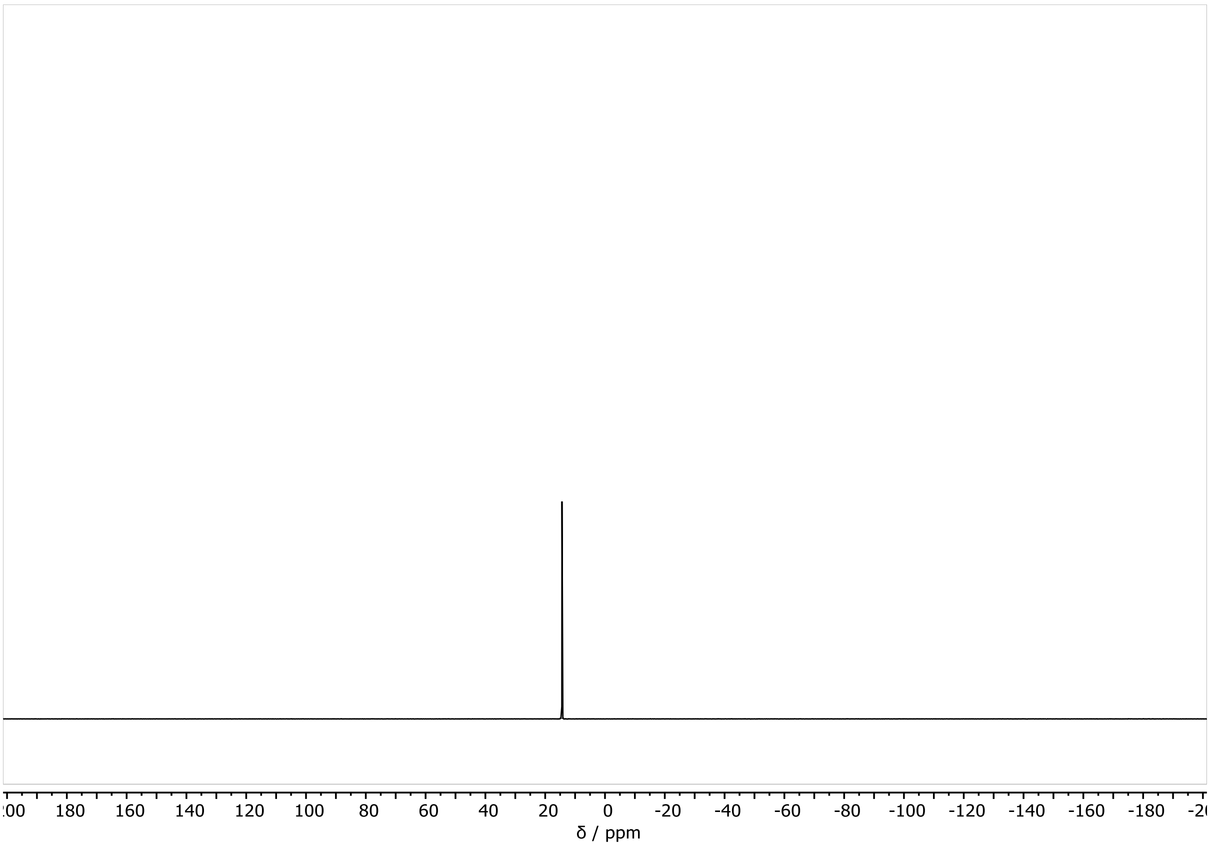

Figure S10. ^{31}P NMR (202 MHz, DMSO-d_6) of **3**

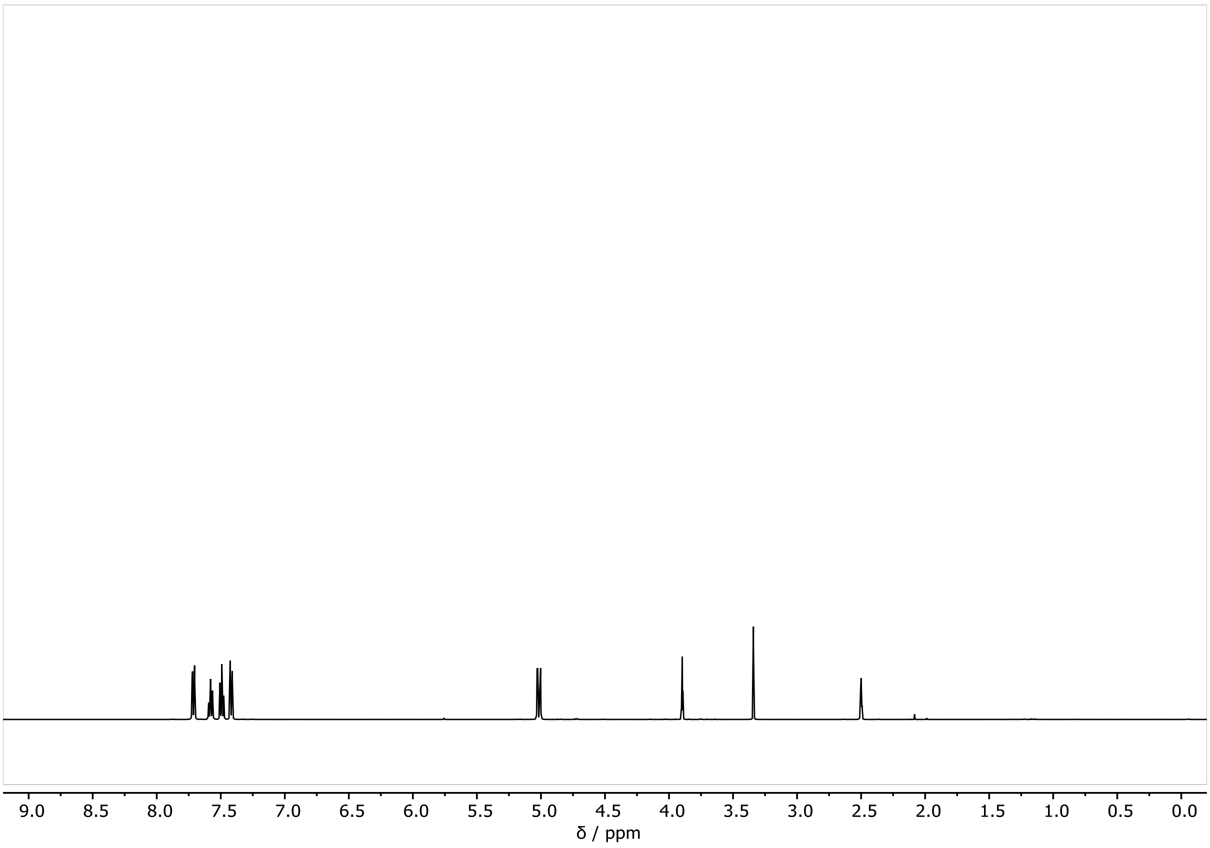

Figure S11. ^{1}H NMR (500 MHz, DMSO-d_6) of **5**

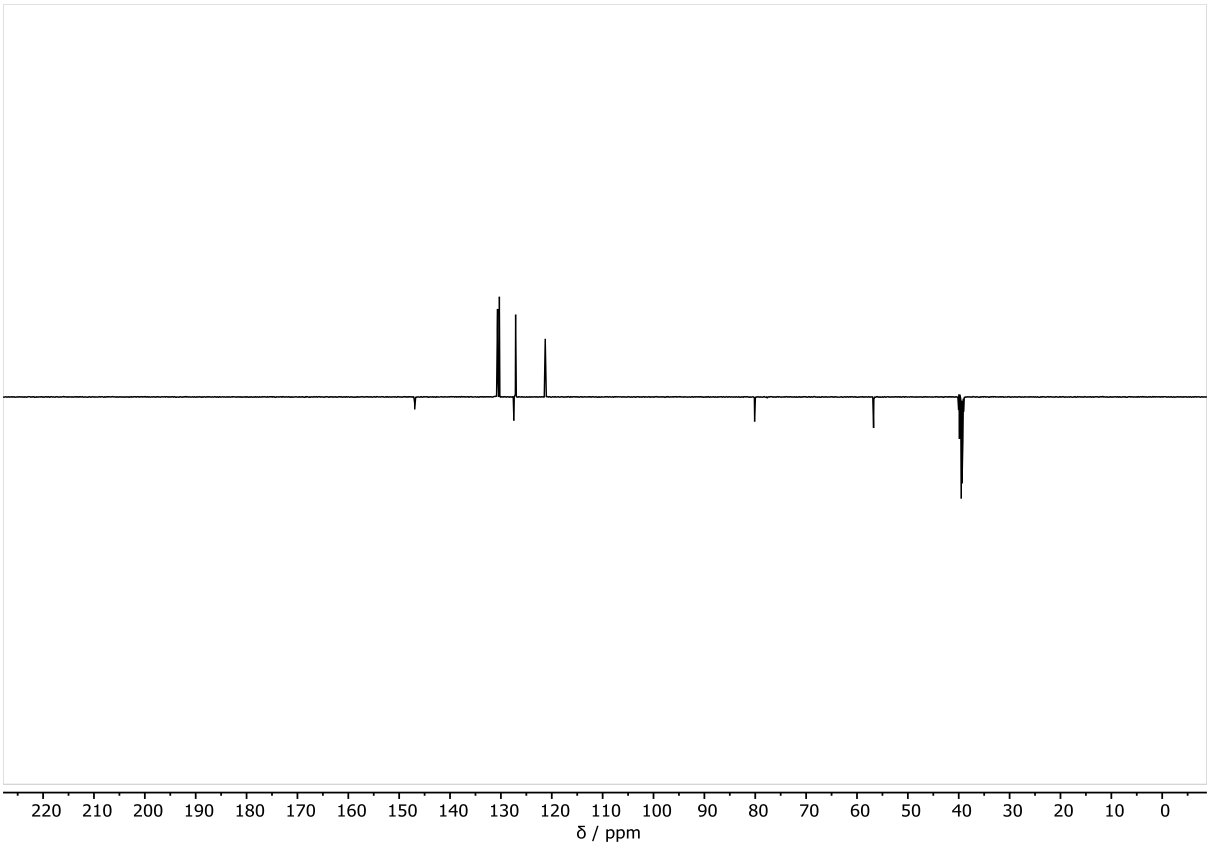

Figure S12. ^{13}C NMR (126 MHz, DMSO-d$_6$) of **5**

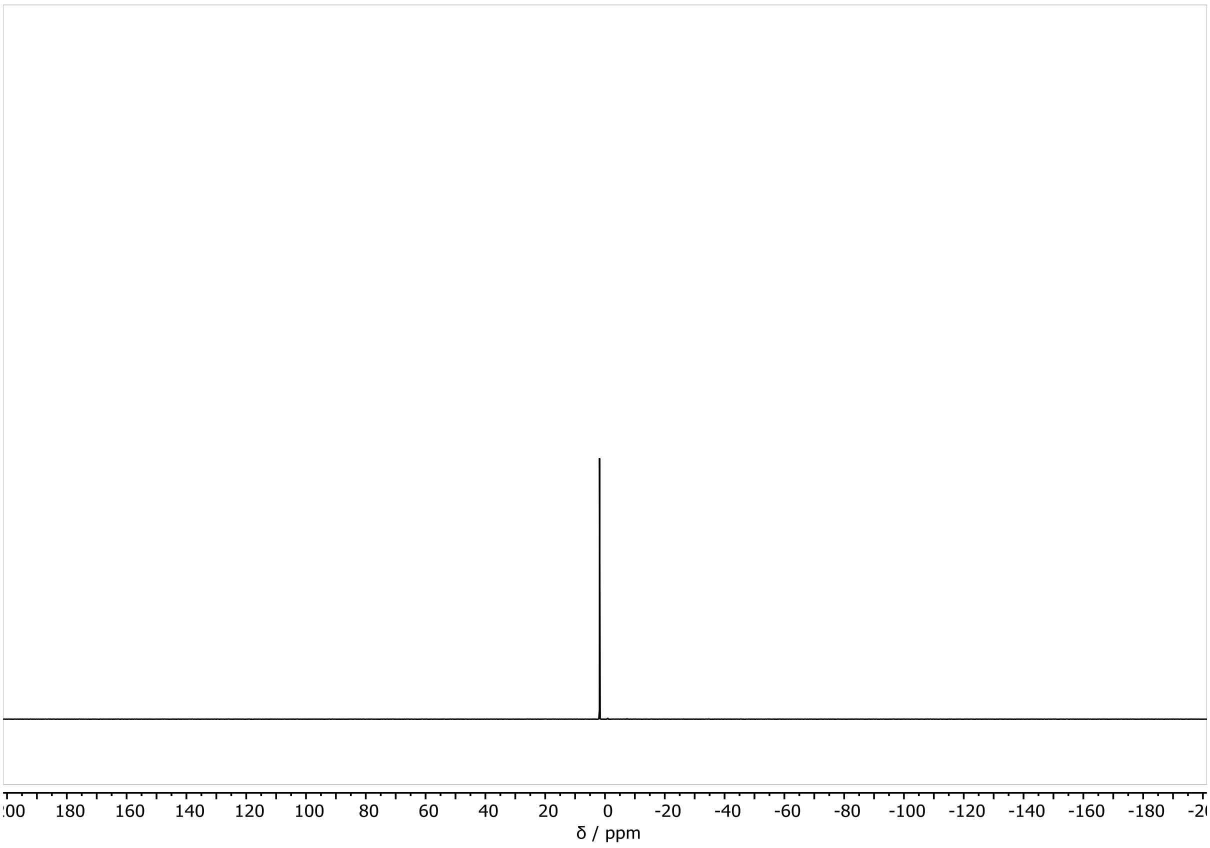

Figure S13. ^{31}P NMR (202 MHz, DMSO-d$_6$) of **5**

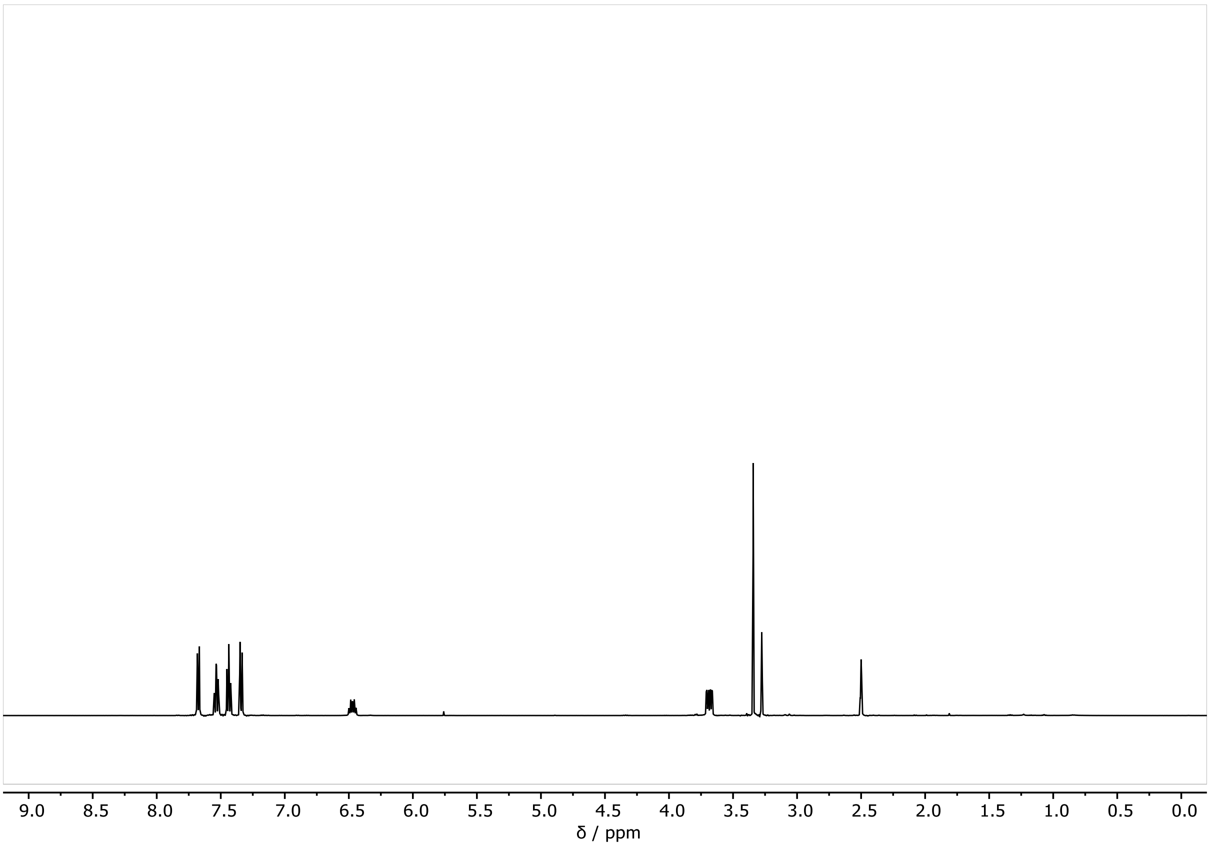

Figure S14. ^{1}H NMR (500 MHz, DMSO-d_6) of **6**

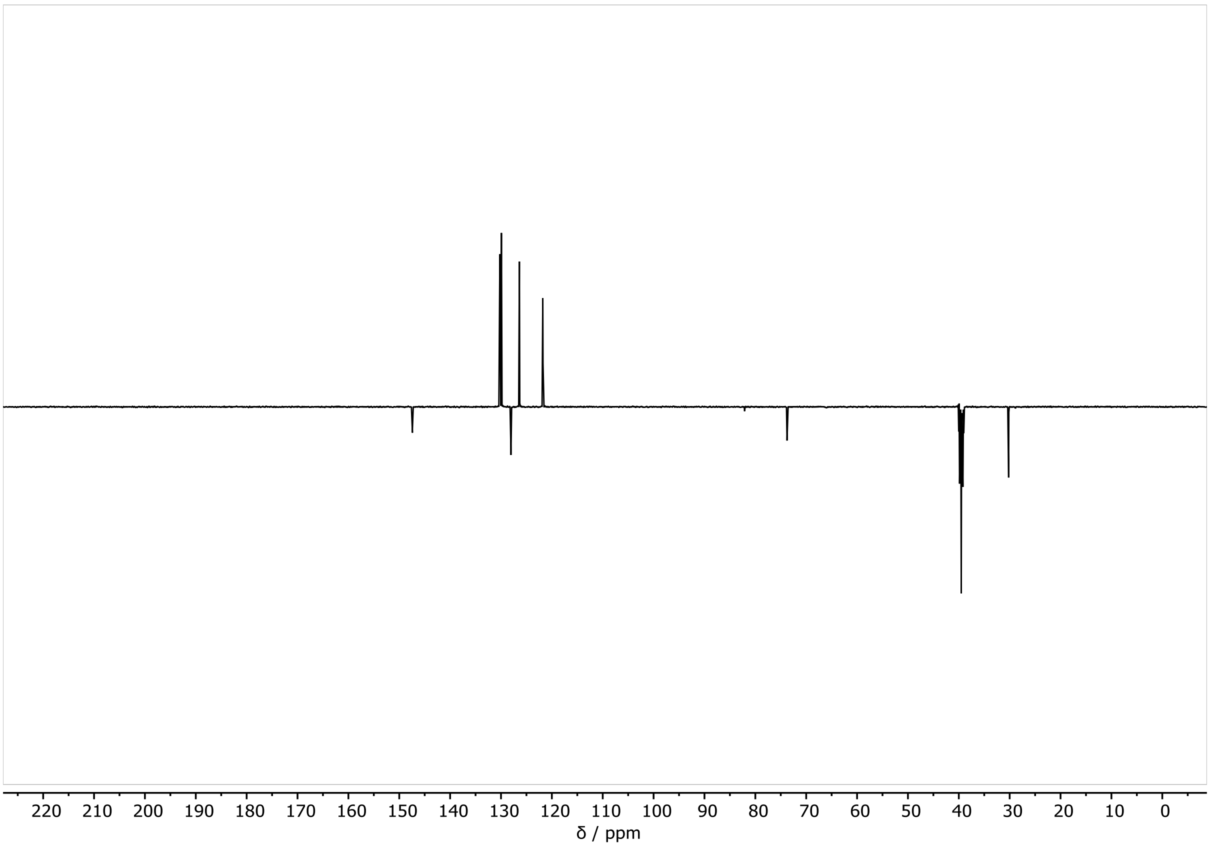

Figure S15. ^{13}C NMR (126 MHz, DMSO-d_6) of **6**

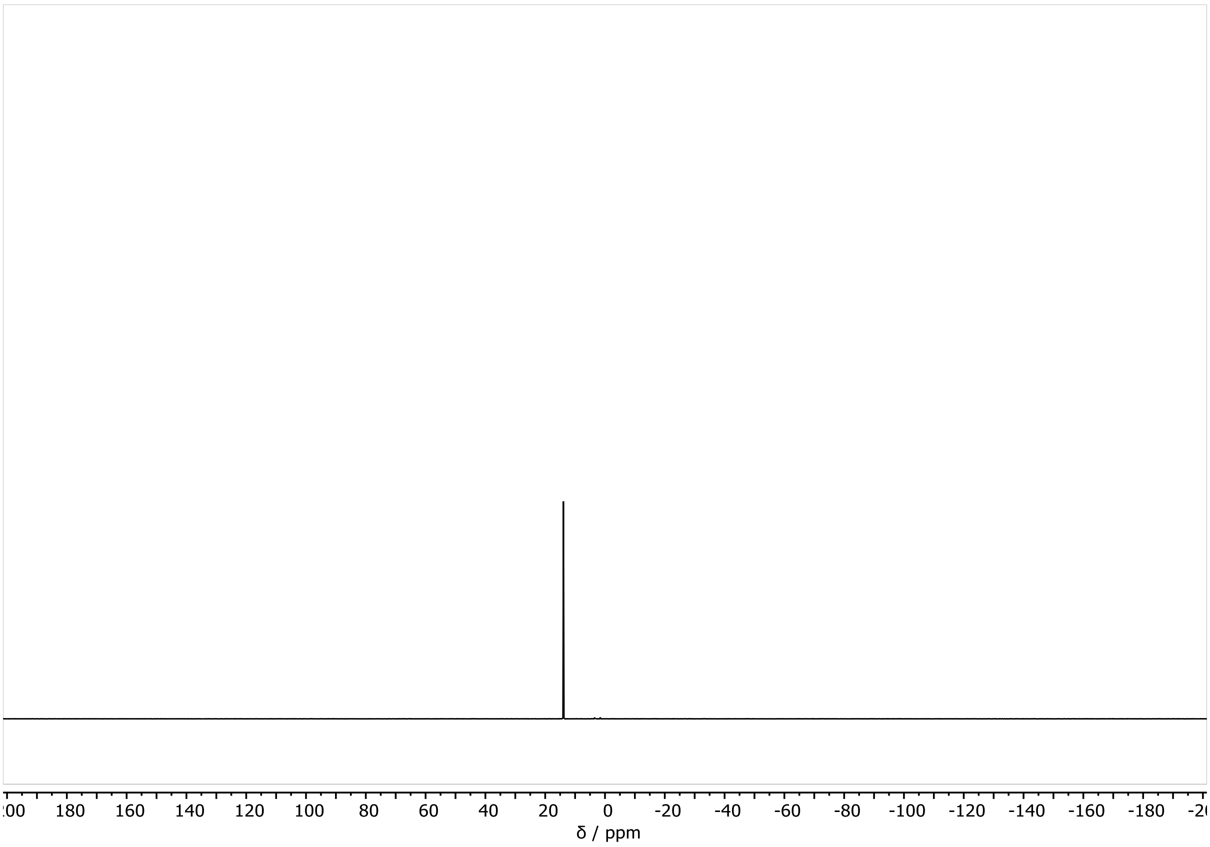

Figure S16. ^{31}P NMR (202 MHz, DMSO-d$_6$) of **6**

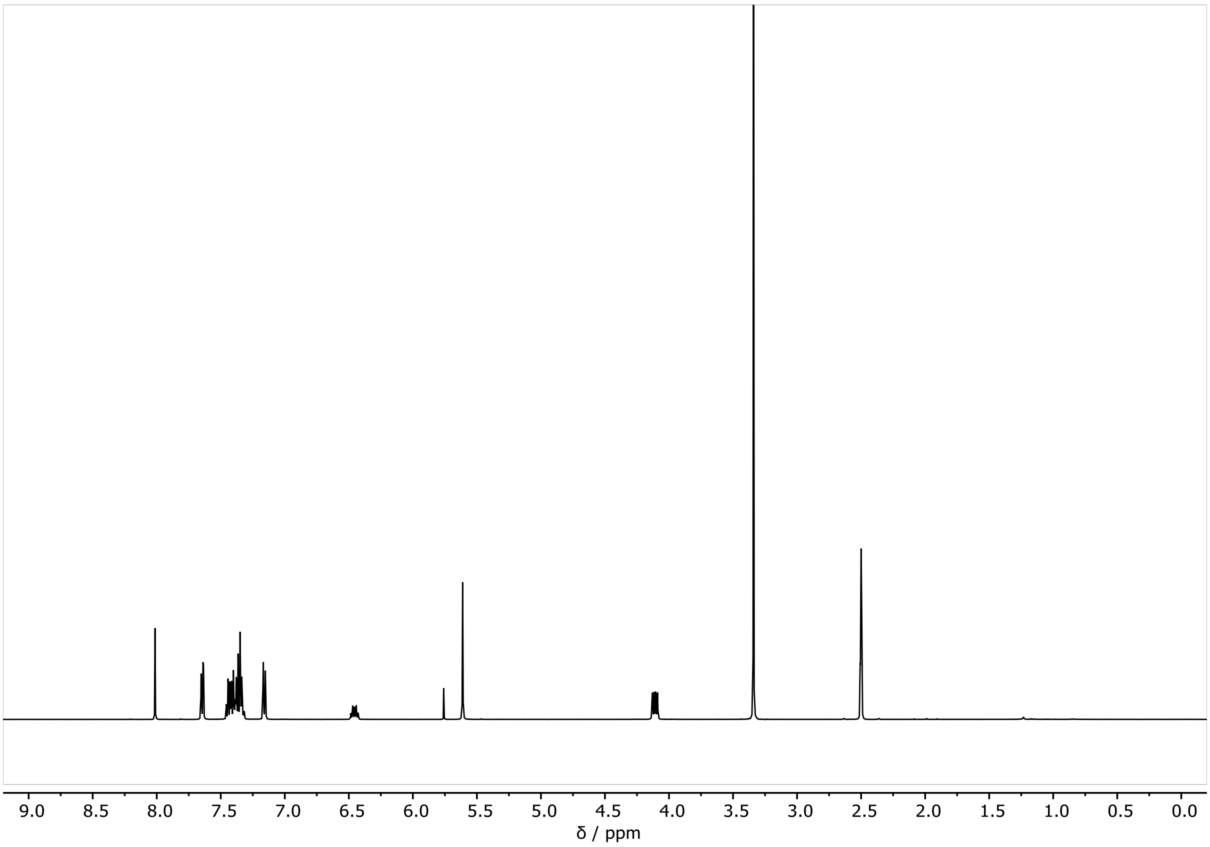

Figure S17. ^{1}H NMR (500 MHz, DMSO-d$_6$) of **12**

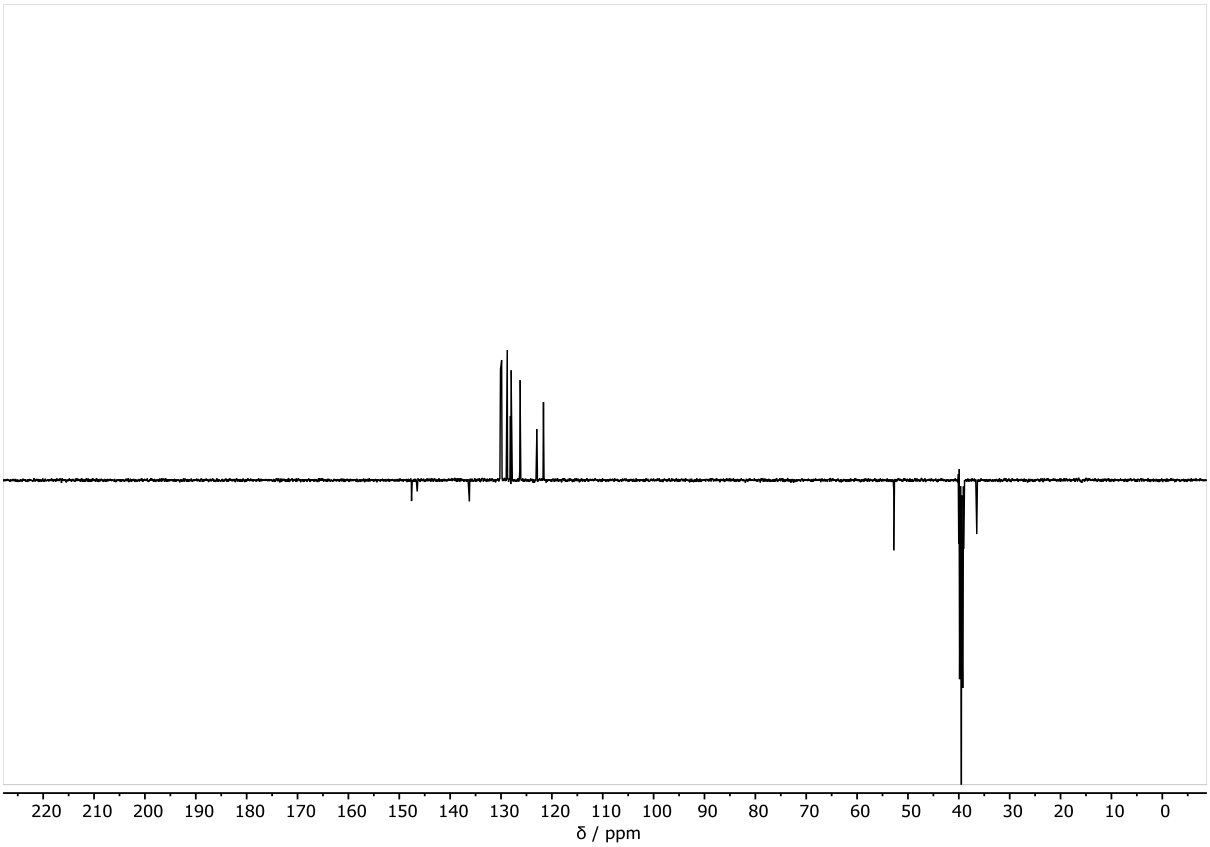

Figure S18. ^{13}C NMR (126 MHz, DMSO-d_6) of **12**

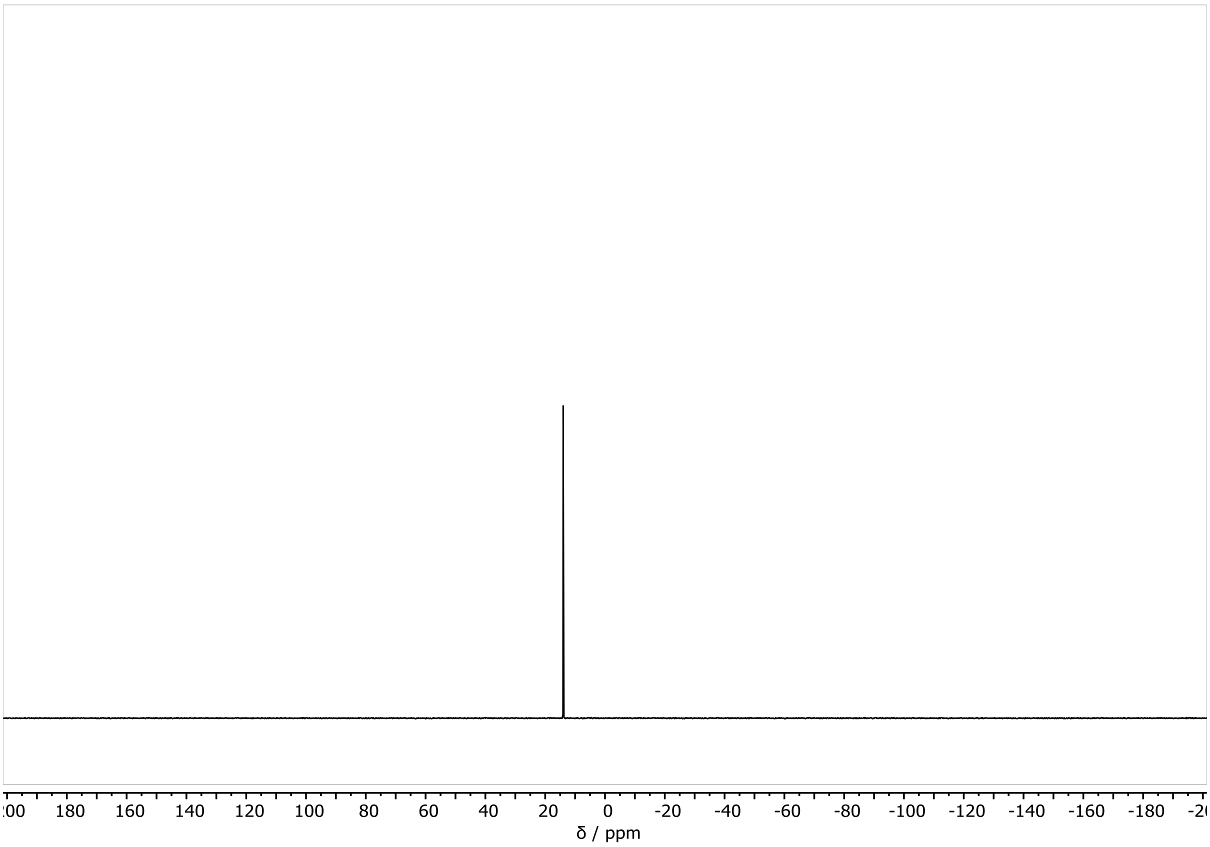

Figure S19. ^{31}P NMR (202 MHz, DMSO-d_6) of **12**

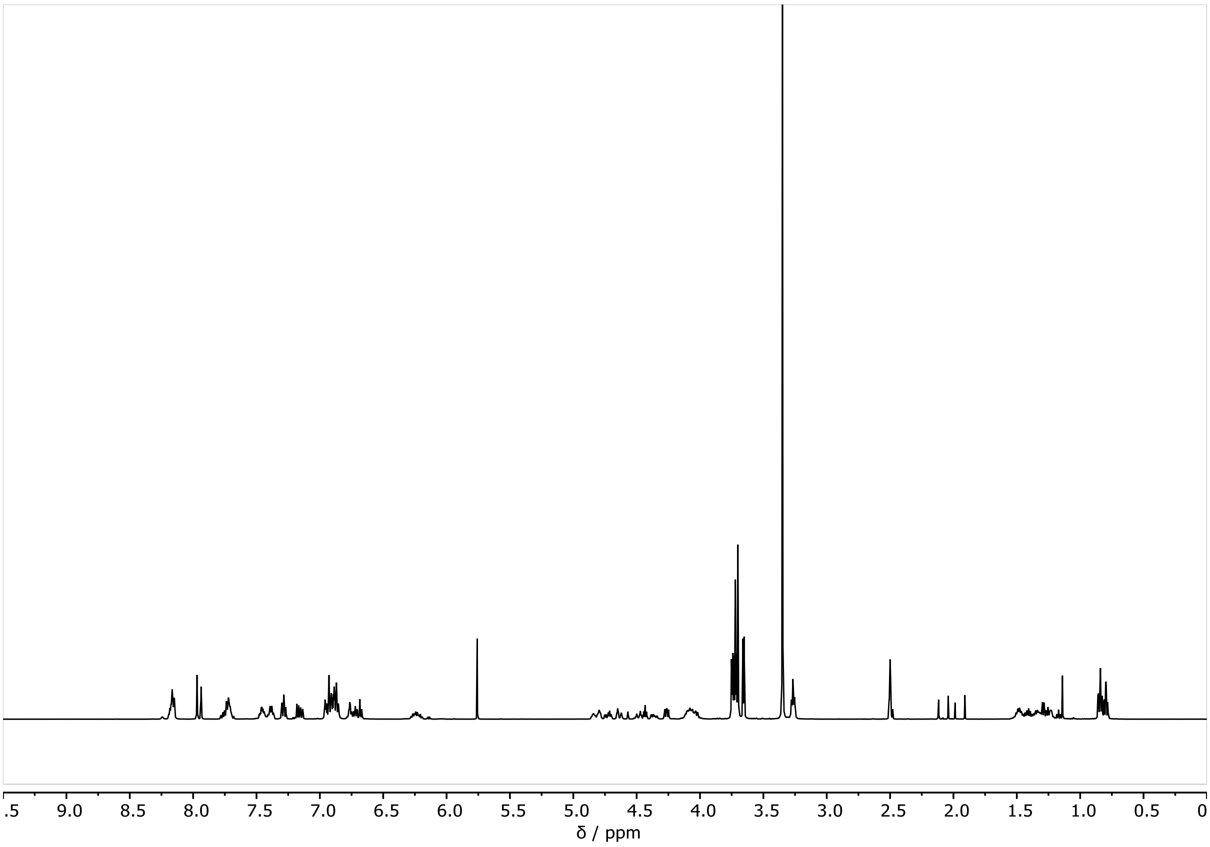

Figure S20. ^{1}H NMR (500 MHz, DMSO-d$_6$) of **9**

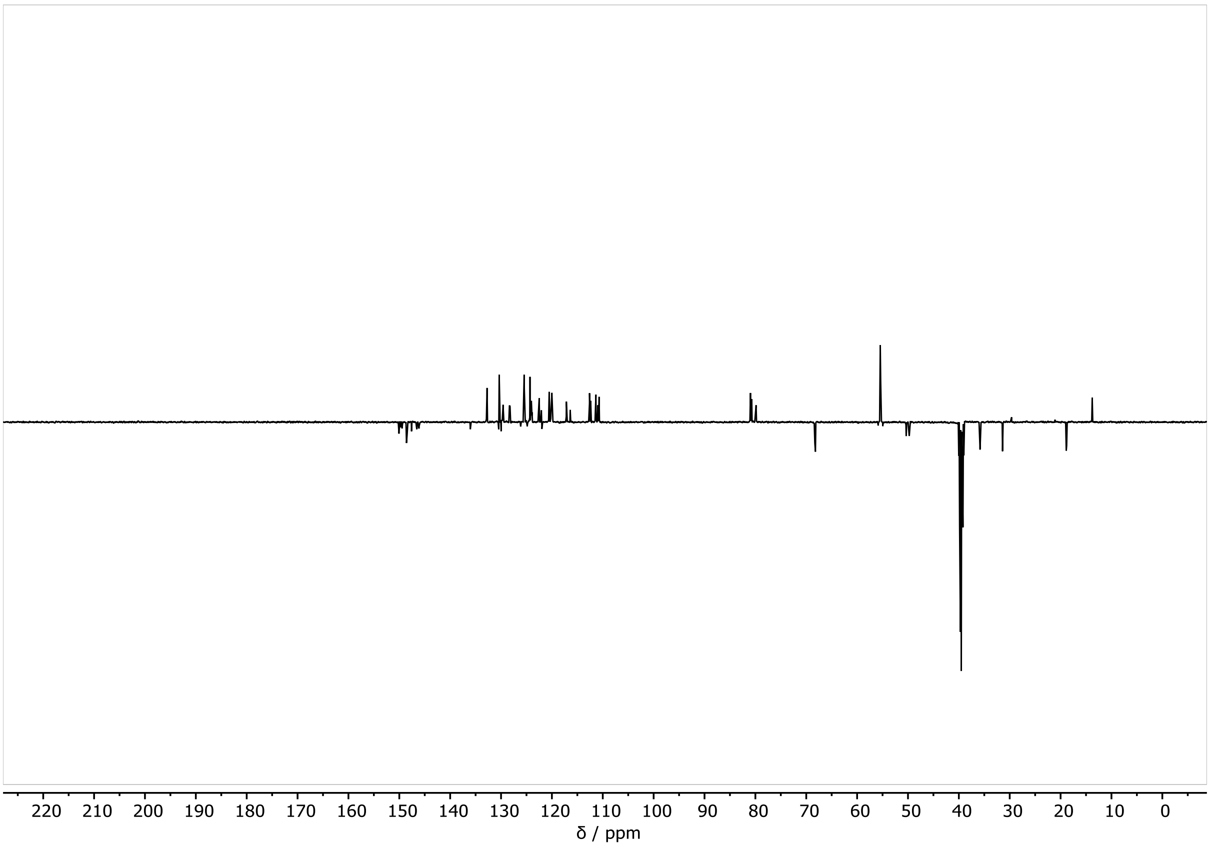

Figure S21. ^{13}C NMR (126 MHz, DMSO-d$_6$) of **9**

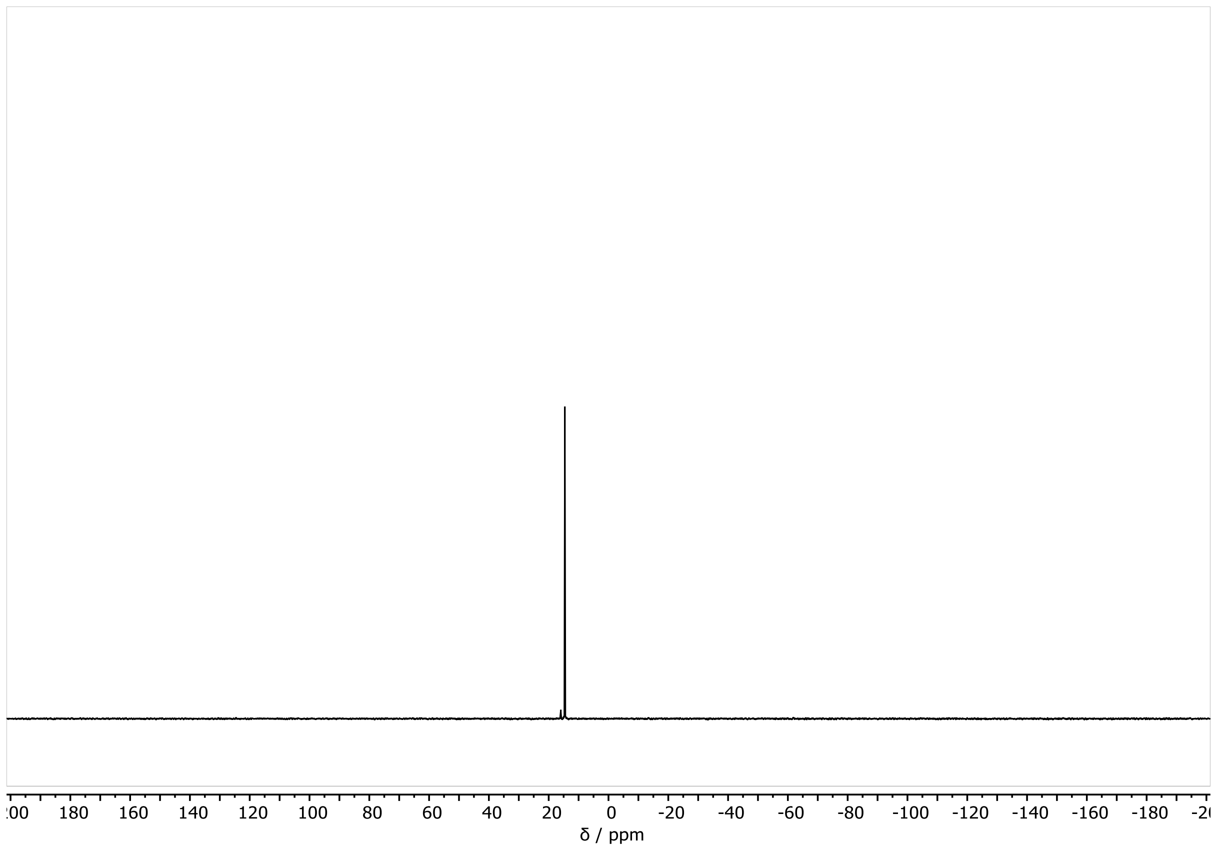

Figure S22. ^{31}P NMR (202 MHz, DMSO-d$_6$) of **9**

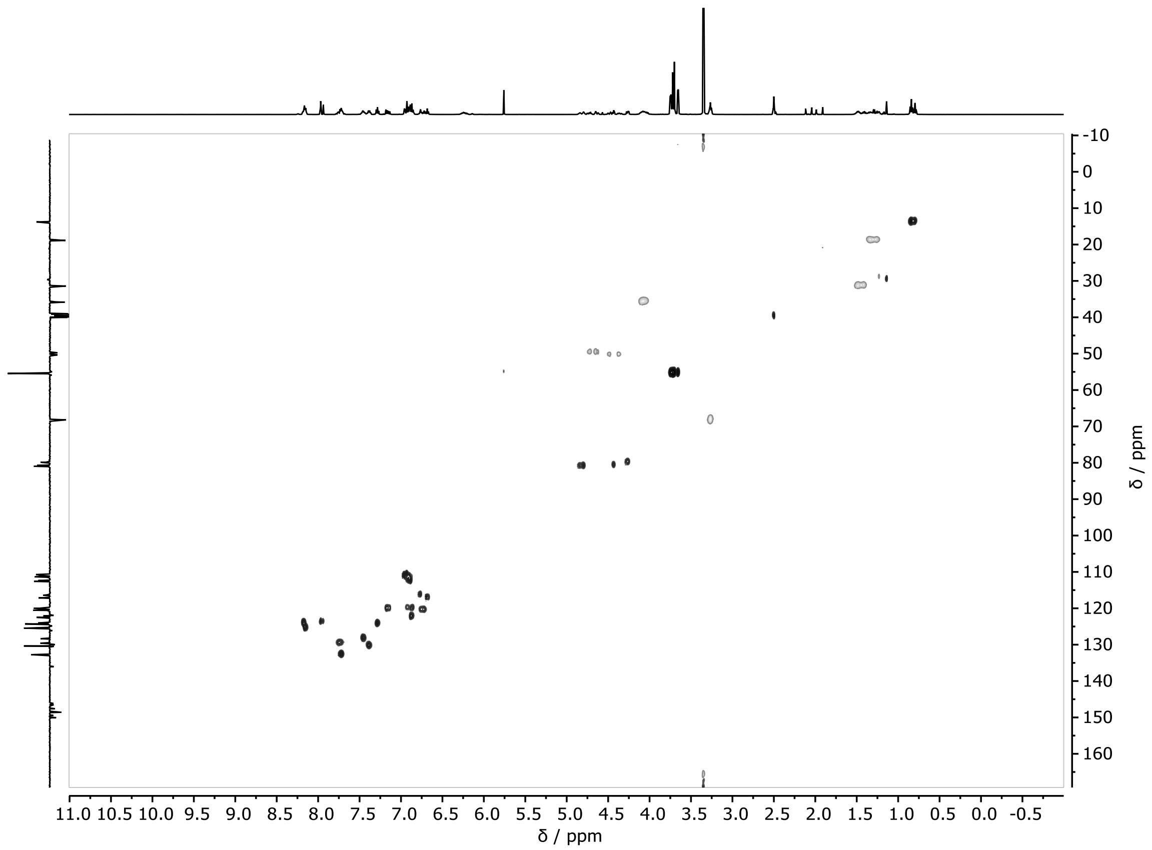

Figure S23. 2D HSQC NMR (500 MHz, DMSO-d$_6$) of **9**

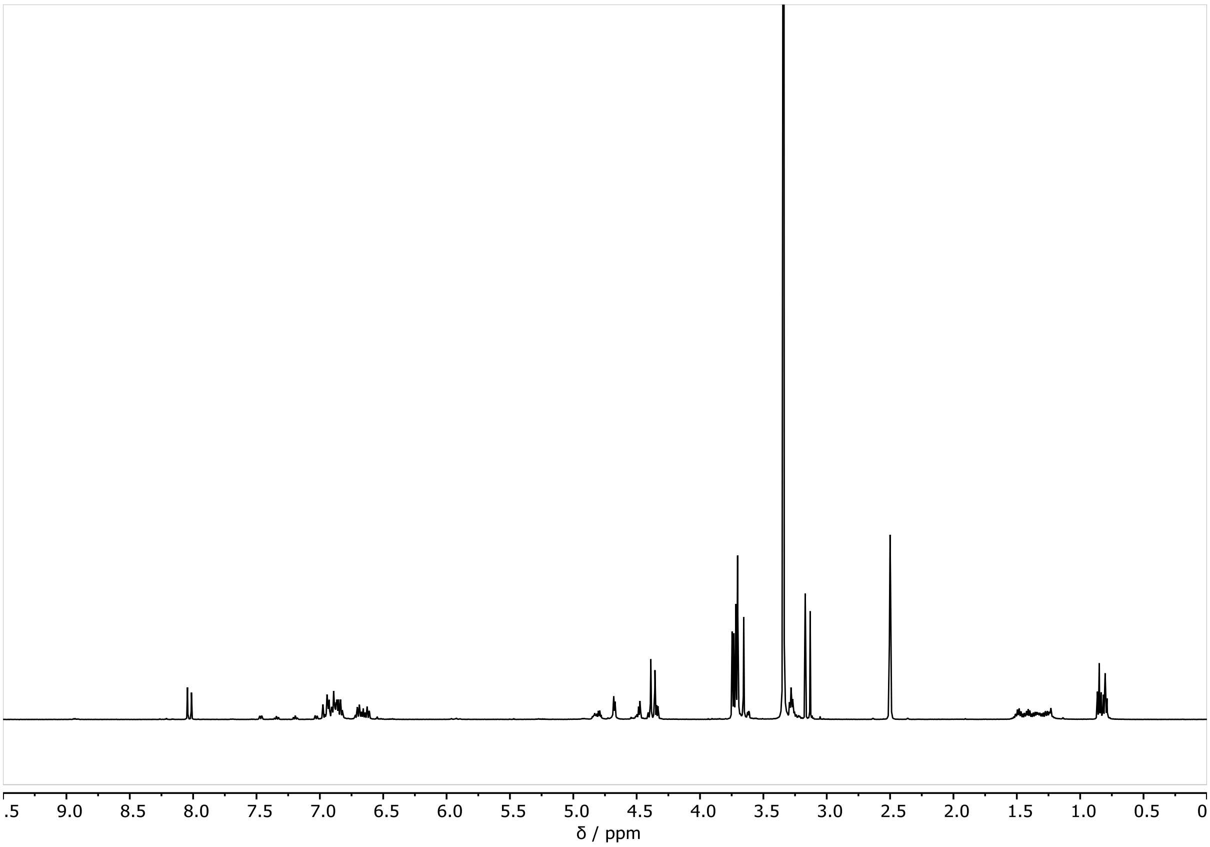

Figure S24. ^{1}H NMR (500 MHz, DMSO-d$_6$) of **10**

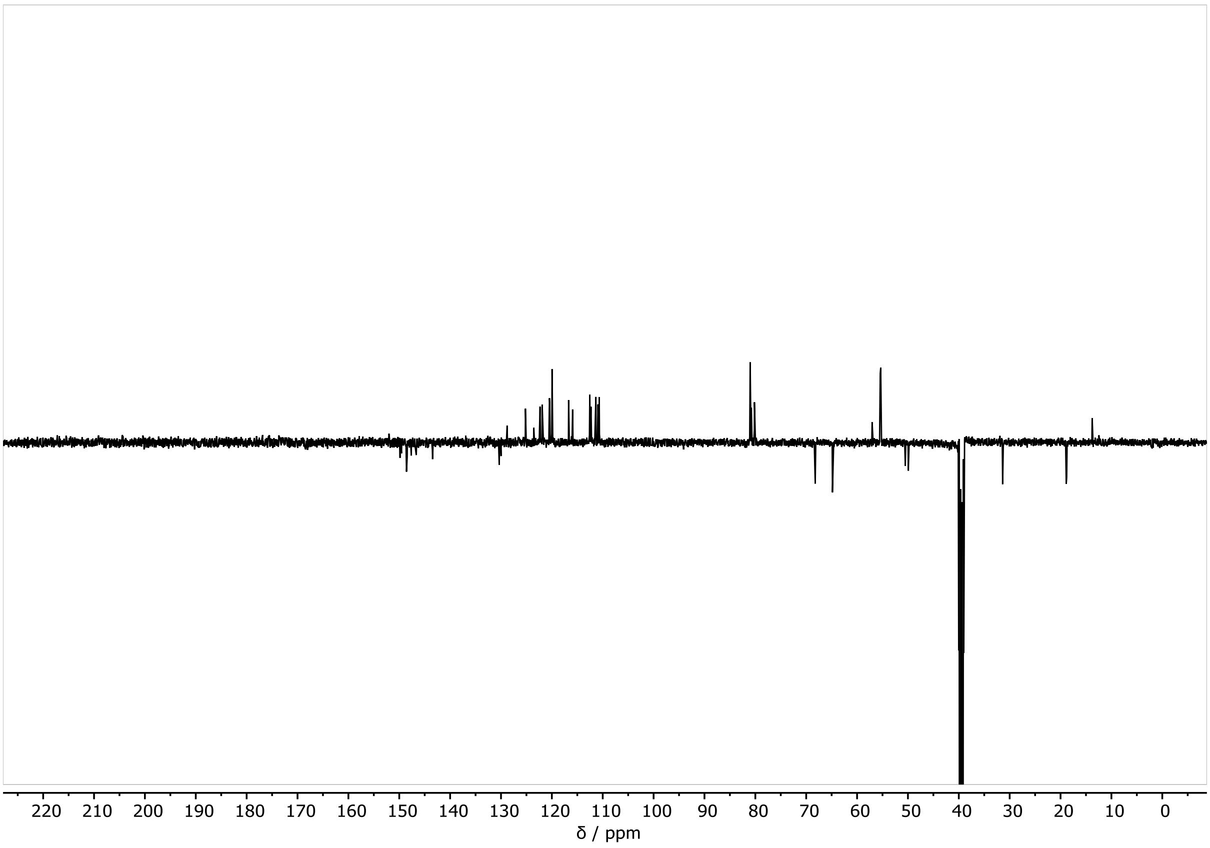

Figure S25. ^{13}C NMR (126 MHz, DMSO-d$_6$) of **10**

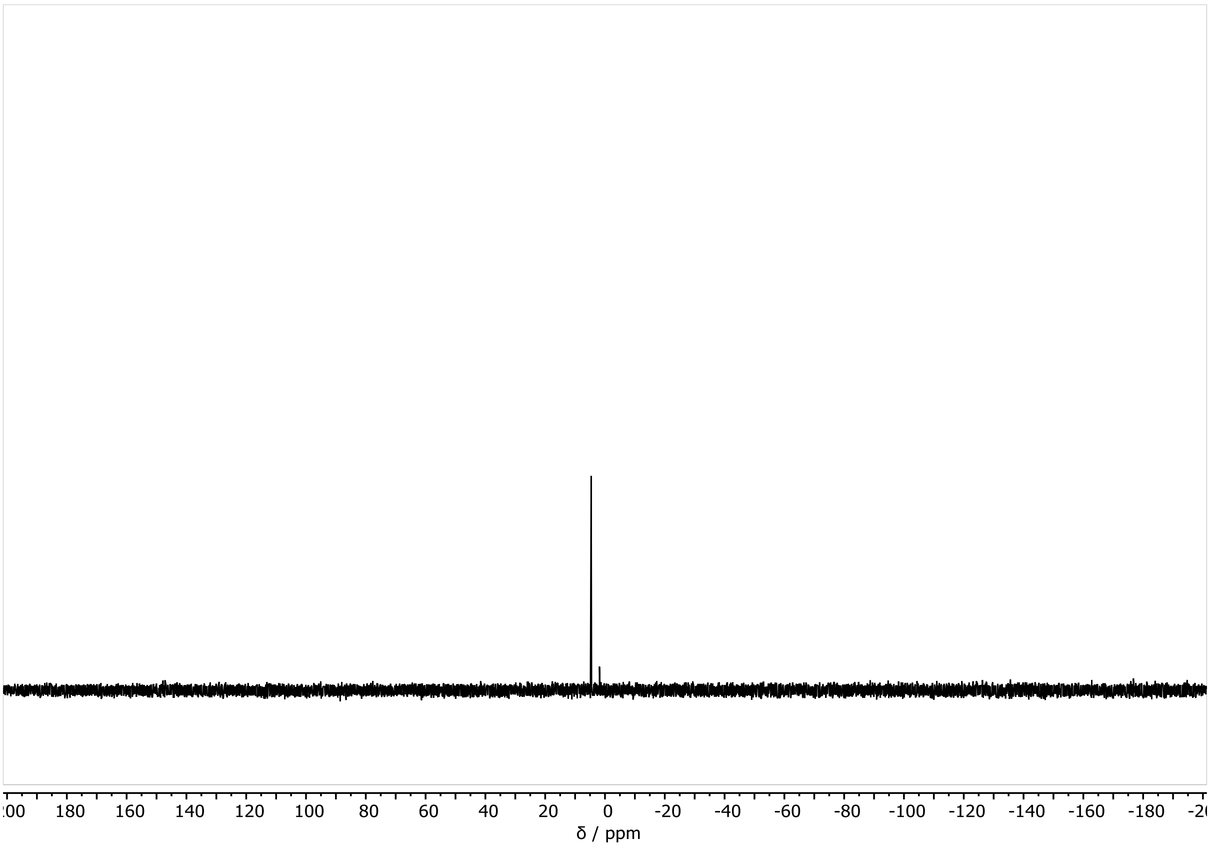

Figure S26. ^{31}P NMR (202 MHz, DMSO-d$_6$) of **10**

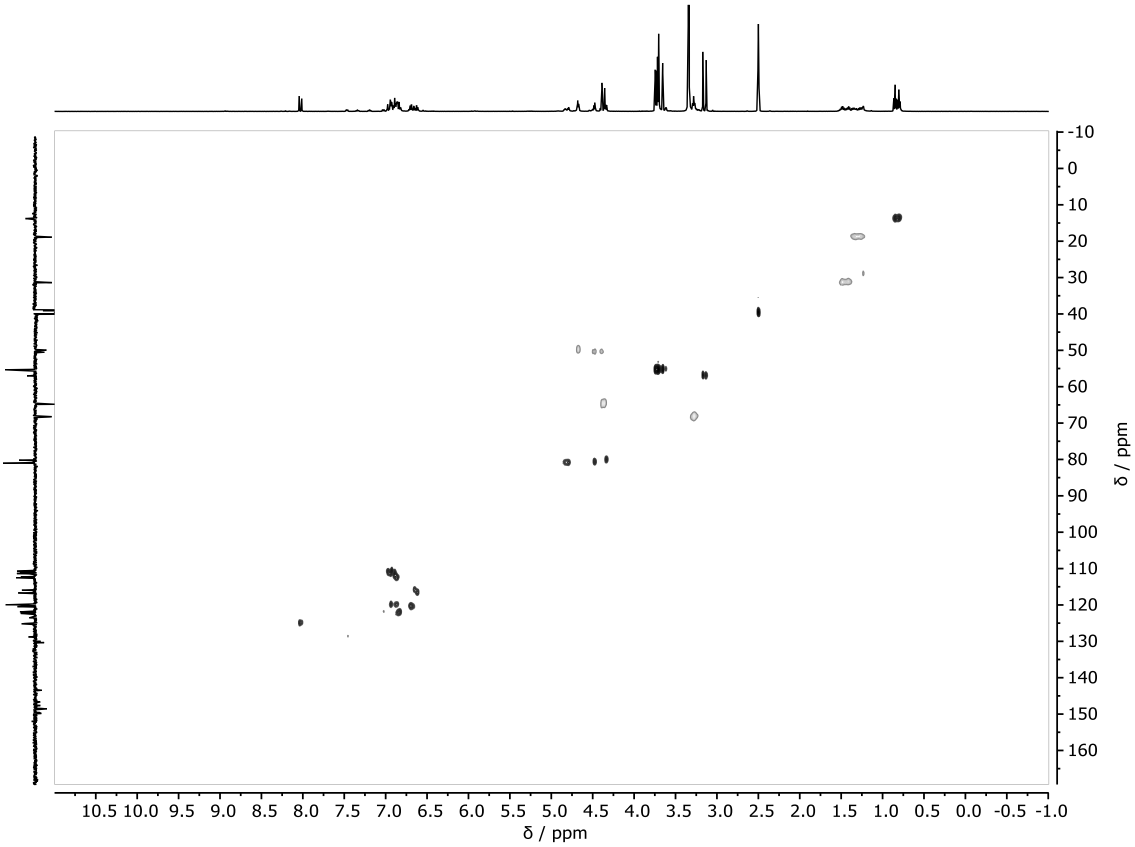

Figure S27. 2D HSQC NMR (500 MHz, DMSO-d$_6$) of **10**

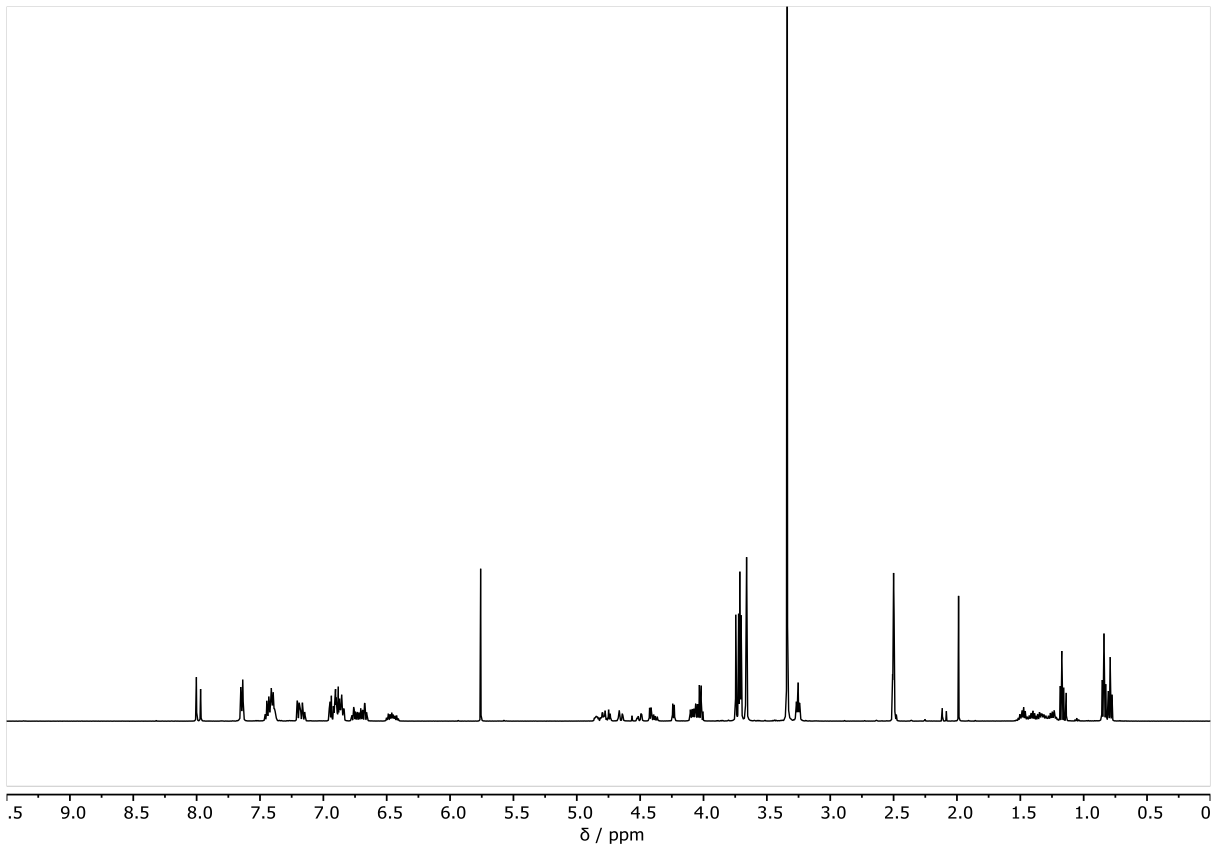

Figure S28. ^{1}H NMR (500 MHz, DMSO-d$_6$) of **11**

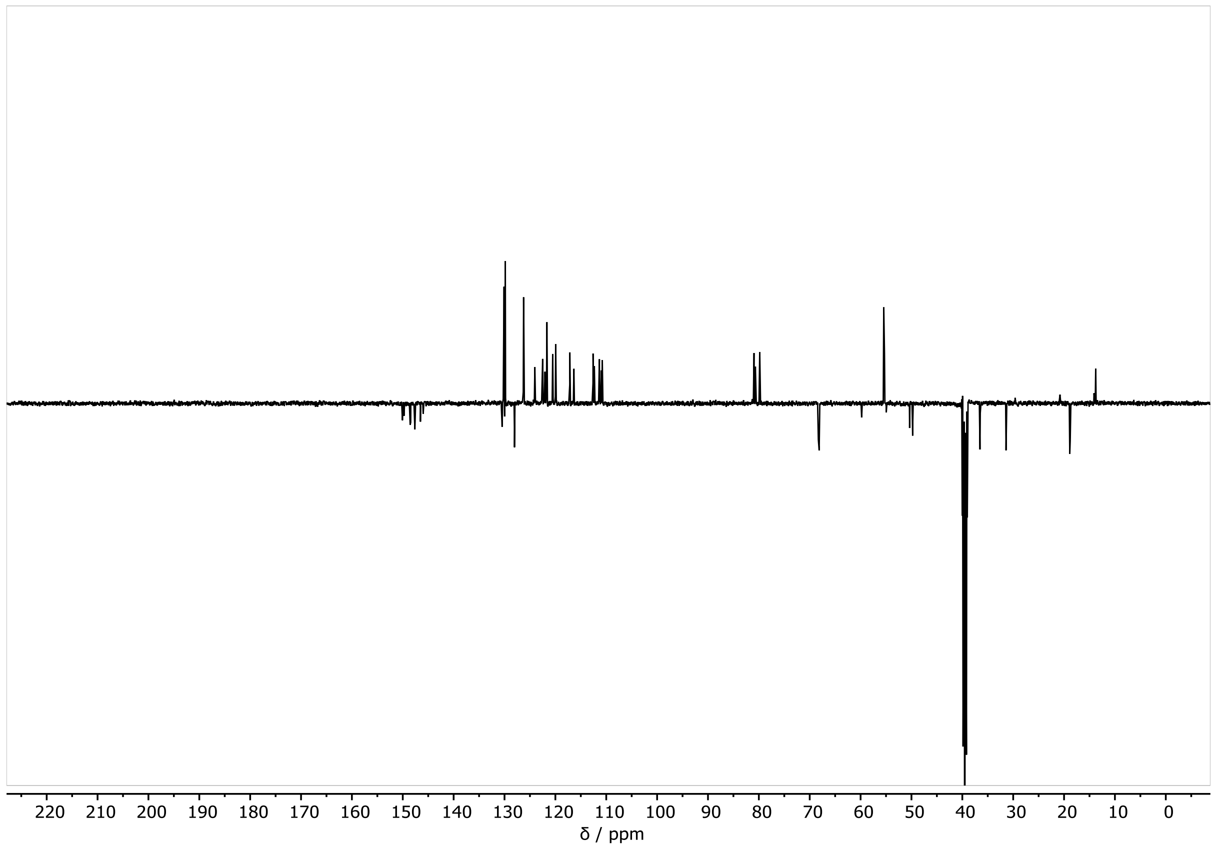

Figure S29. ^{13}C NMR (126 MHz, DMSO-d$_6$) of **11**

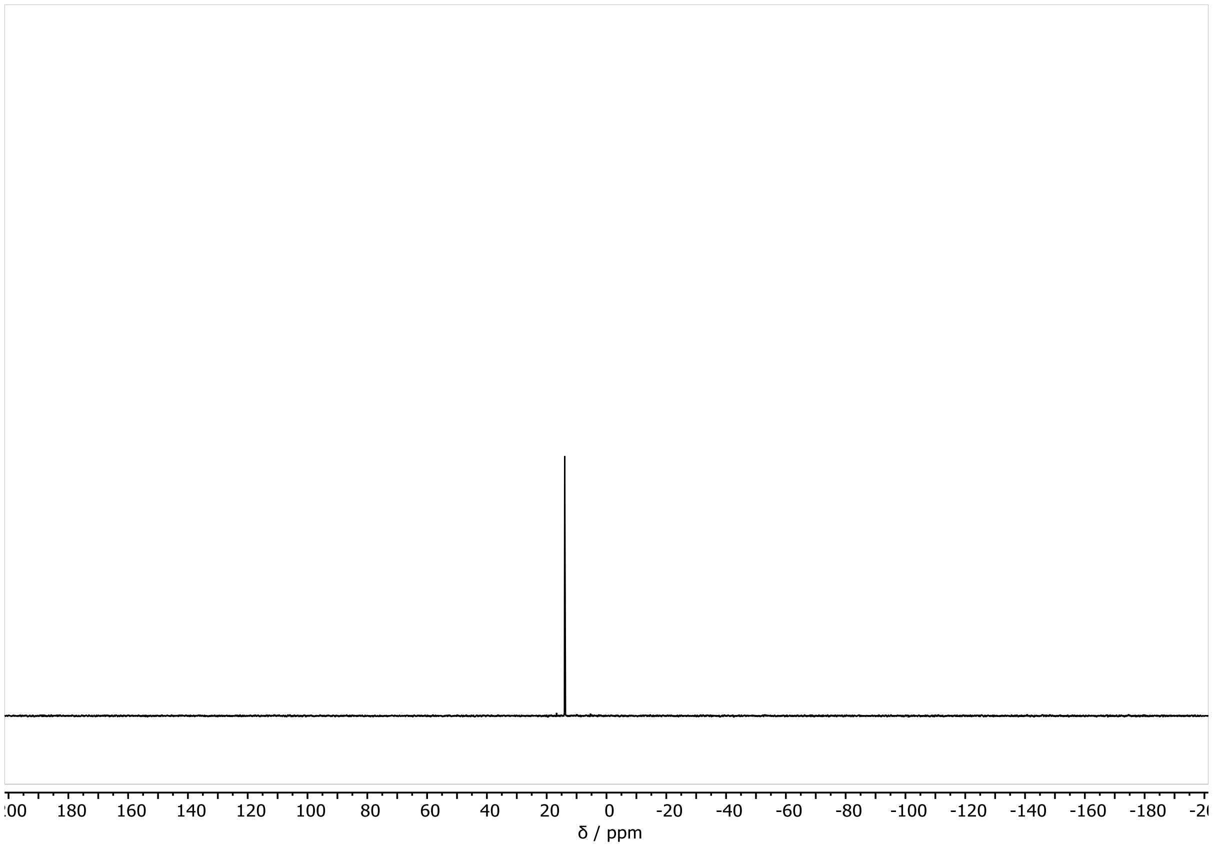

Figure S30. ^{31}P NMR (202 MHz, DMSO-d$_6$) of **11**

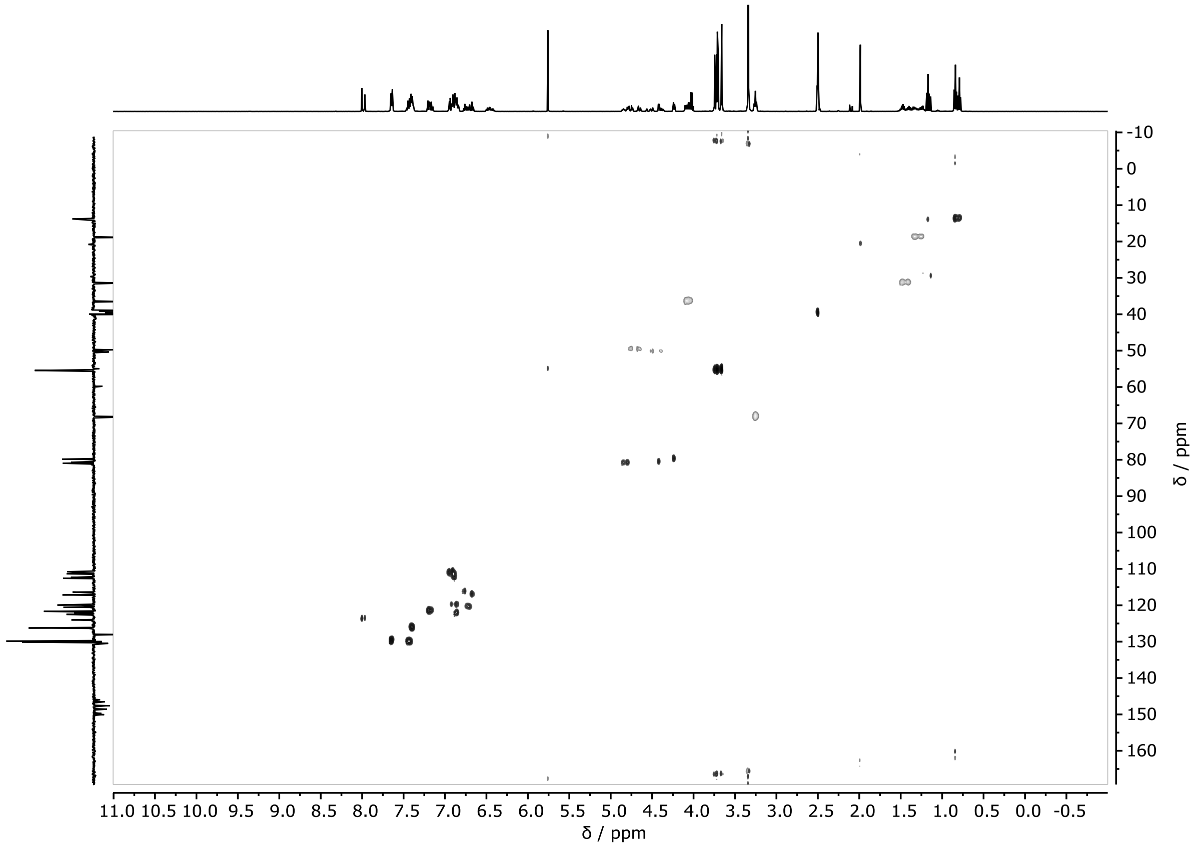

Figure S31. 2D HSQC NMR (500 MHz, DMSO-d$_6$) of **11**

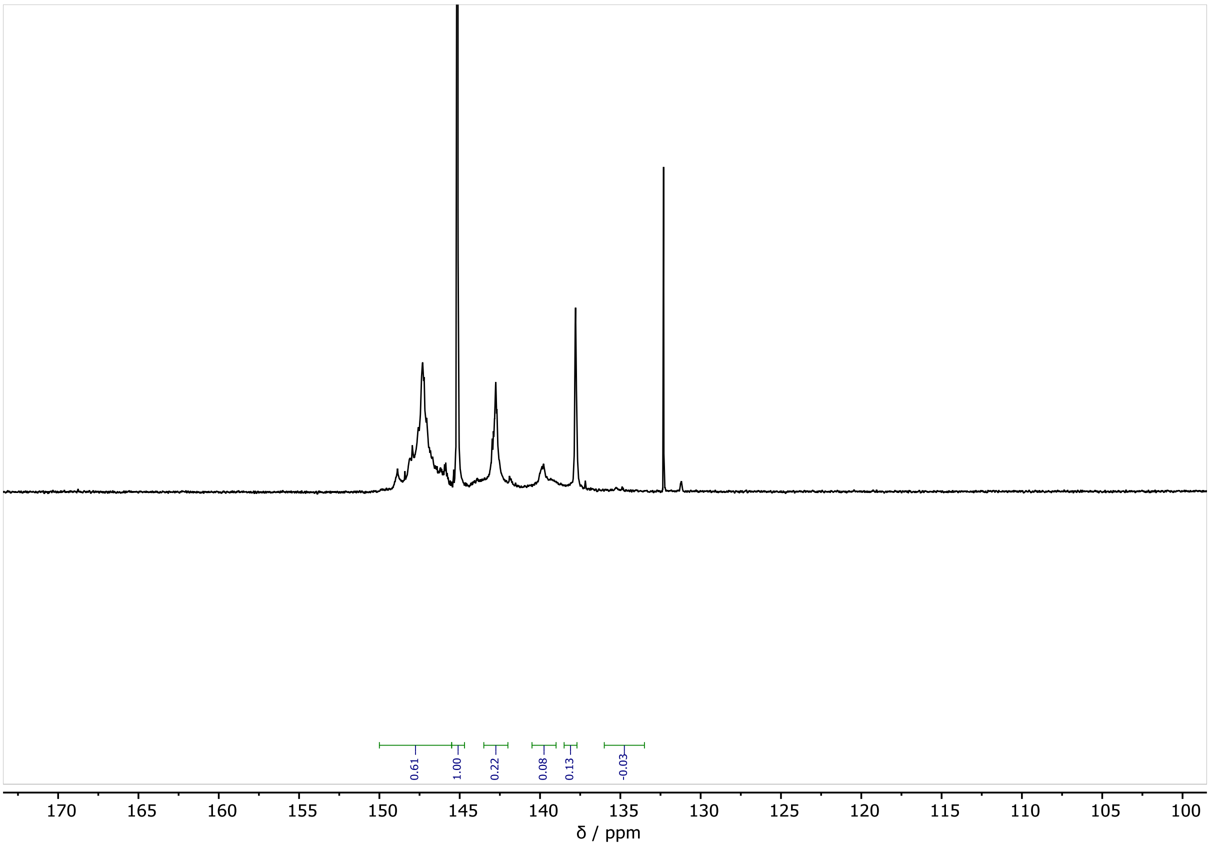

Figure S32. Quantitative ^{31}P NMR (202 MHz, DMSO-d_6) of **DPW Lignin** after phosphitylation

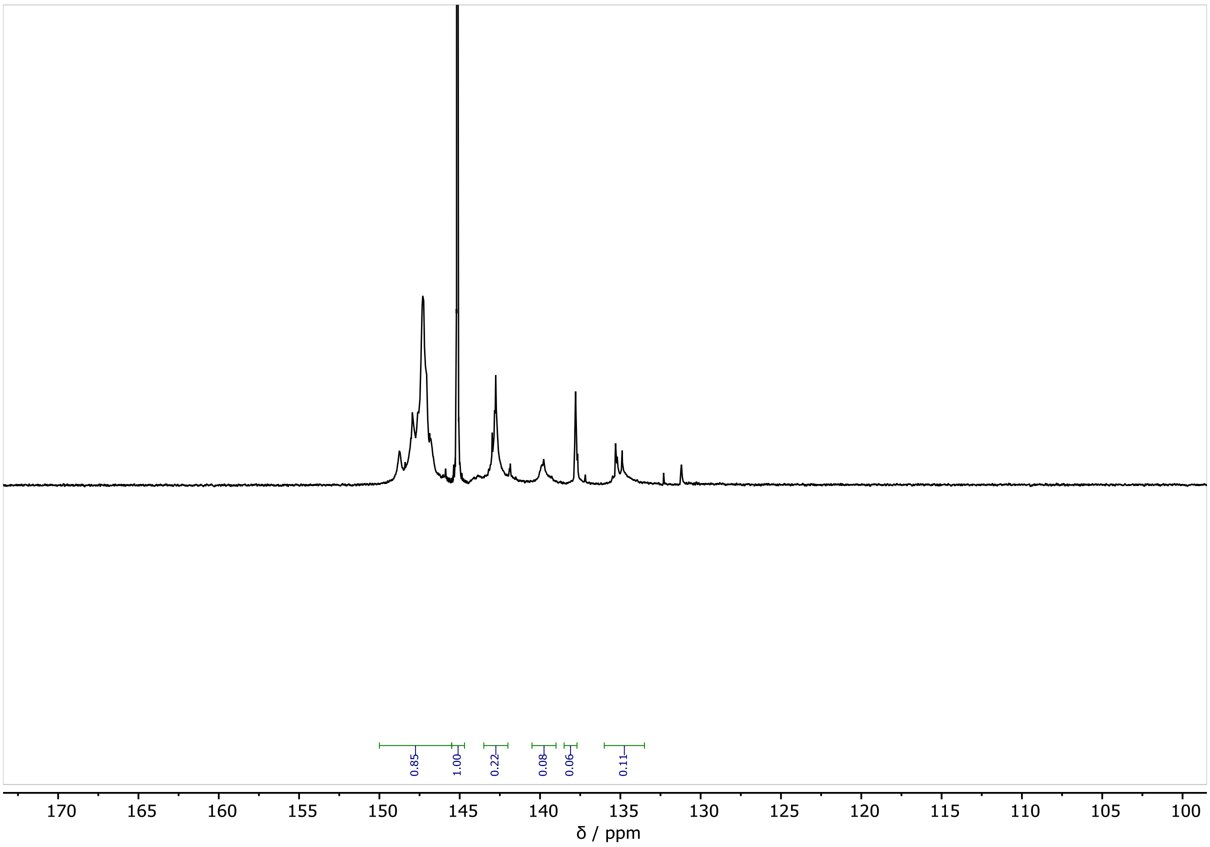

Figure S33. Quantitative ^{31}P NMR (202 MHz, DMSO-d_6) of **DeAcyl Lignin** after phosphitylation

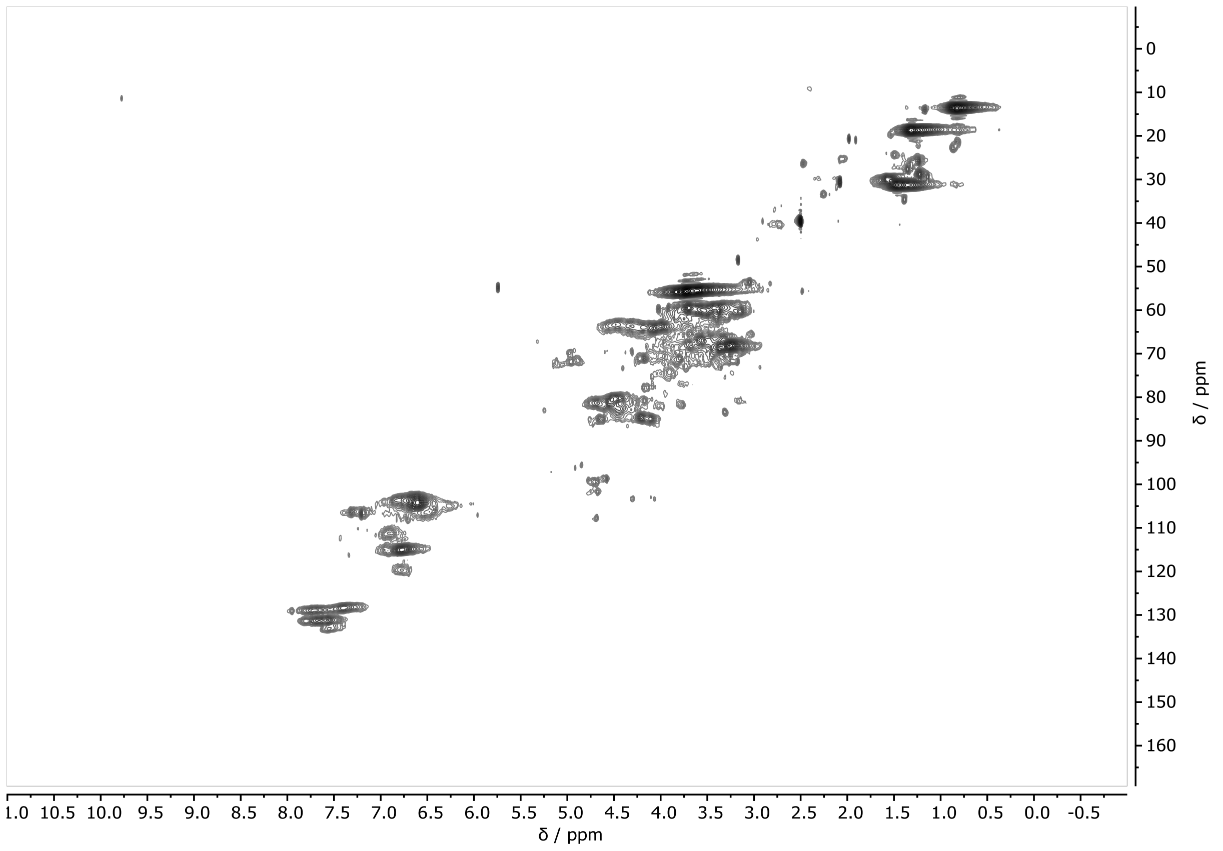

Figure S34. 2D HSQC NMR (700 MHz, DMSO-d_6) of **DPW Lignin**

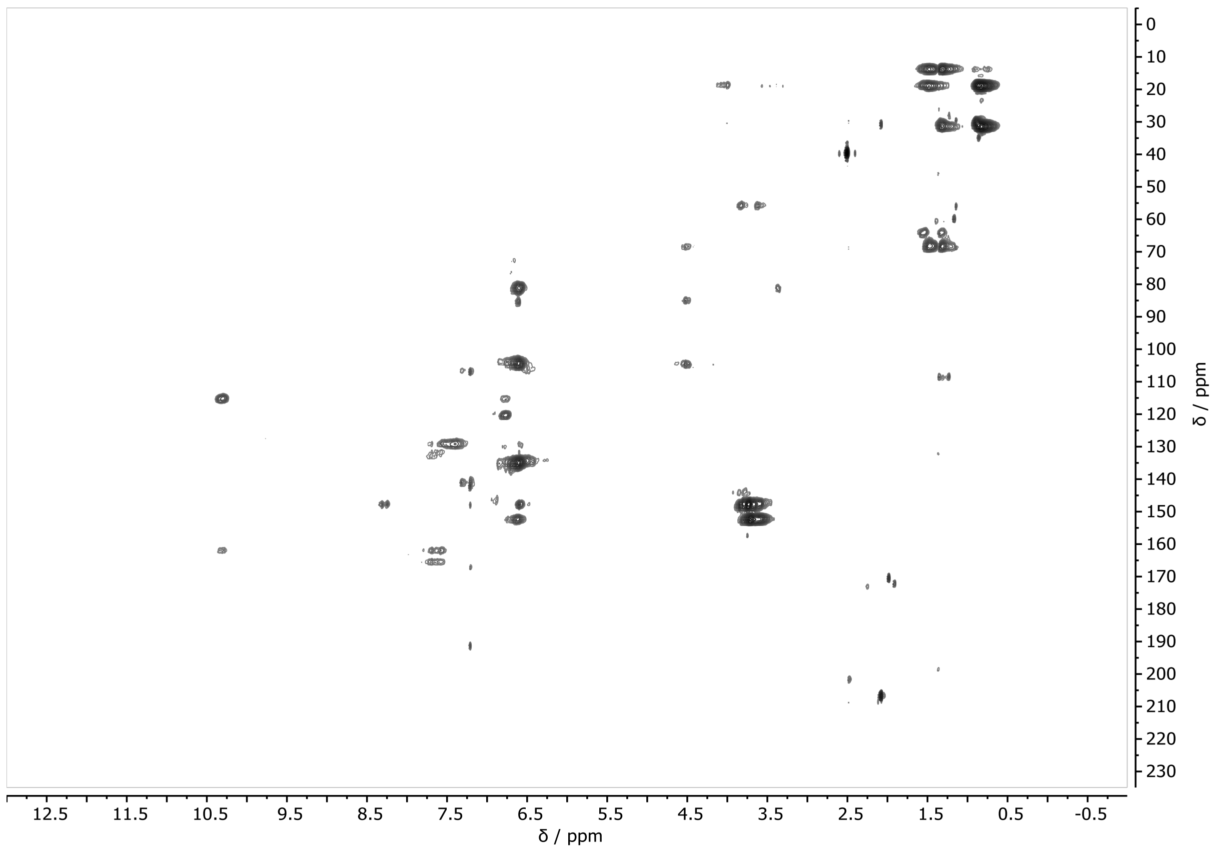

Figure S35. 2D HMBC NMR (700 MHz, DMSO-d_6) of **DPW Lignin**

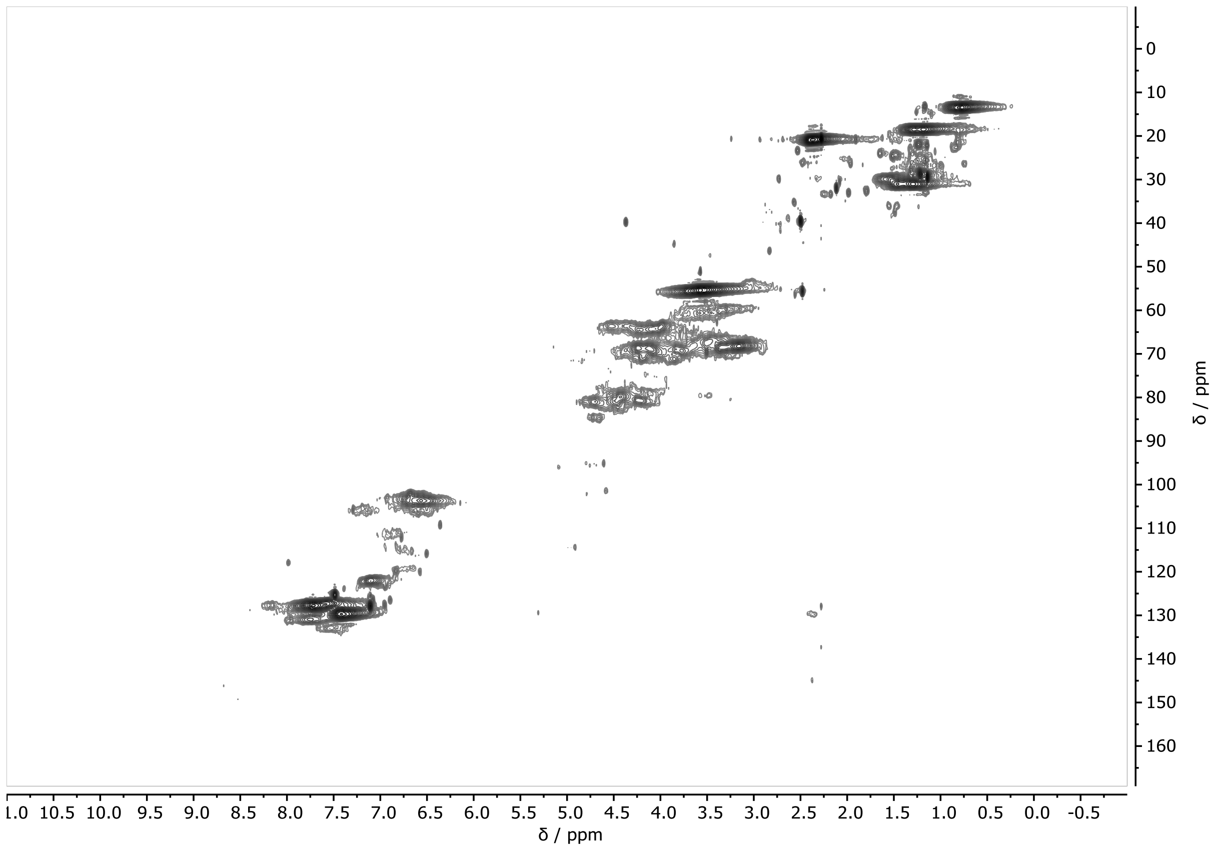

Figure S36. 2D HSQC NMR (700 MHz, DMSO-d6) of **DPW Lignin-Ts**

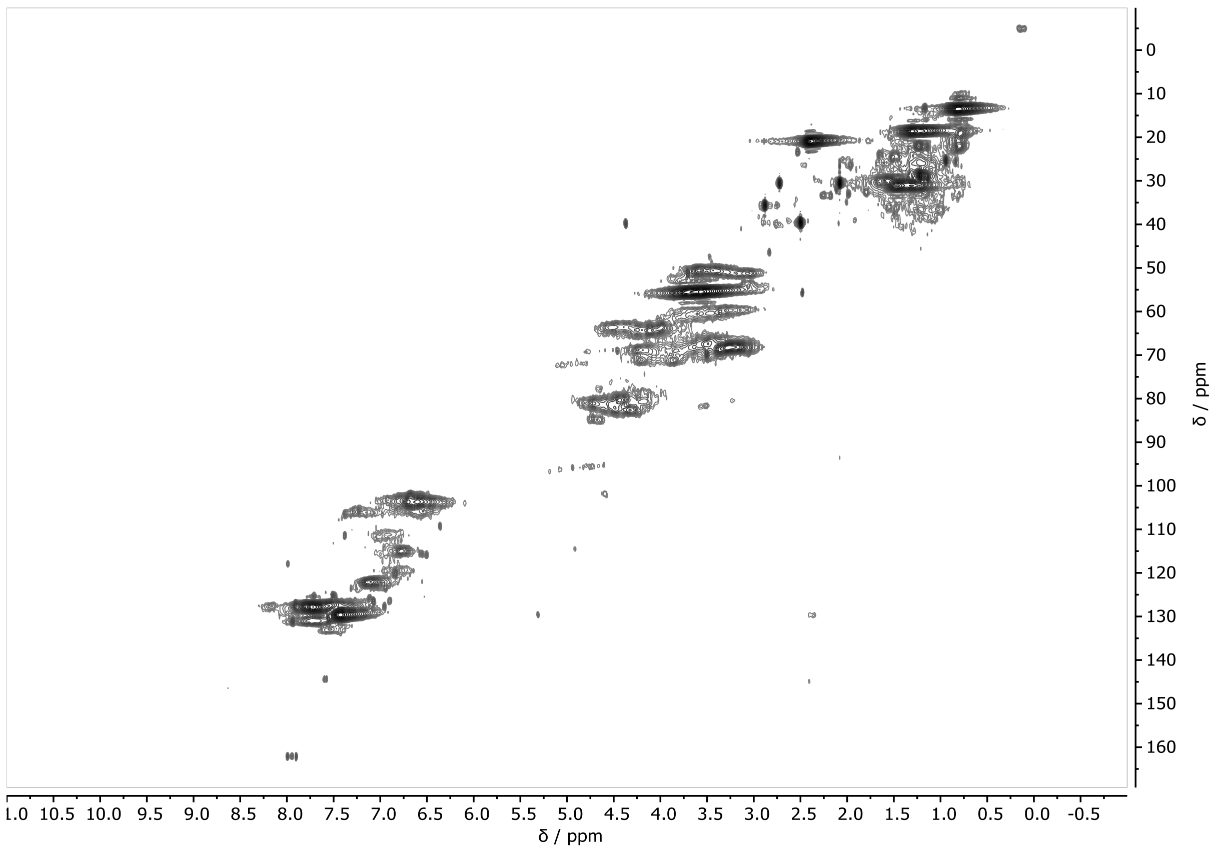

Figure S37. 2D HSQC NMR (700 MHz, DMSO-d6) of **DPW Lignin-N_3**

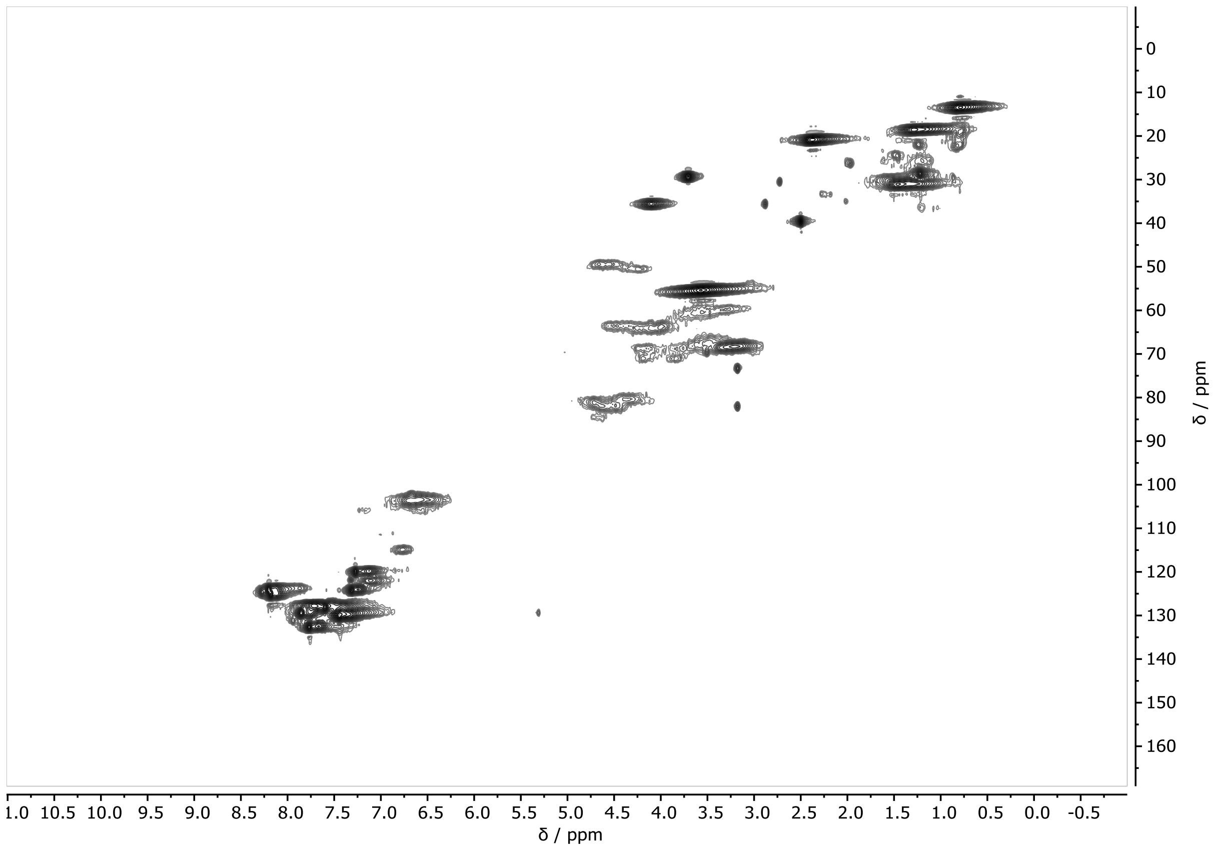

Figure S38. 2D HSQC NMR (700 MHz, DMSO-d$_6$) of **DPW-3**

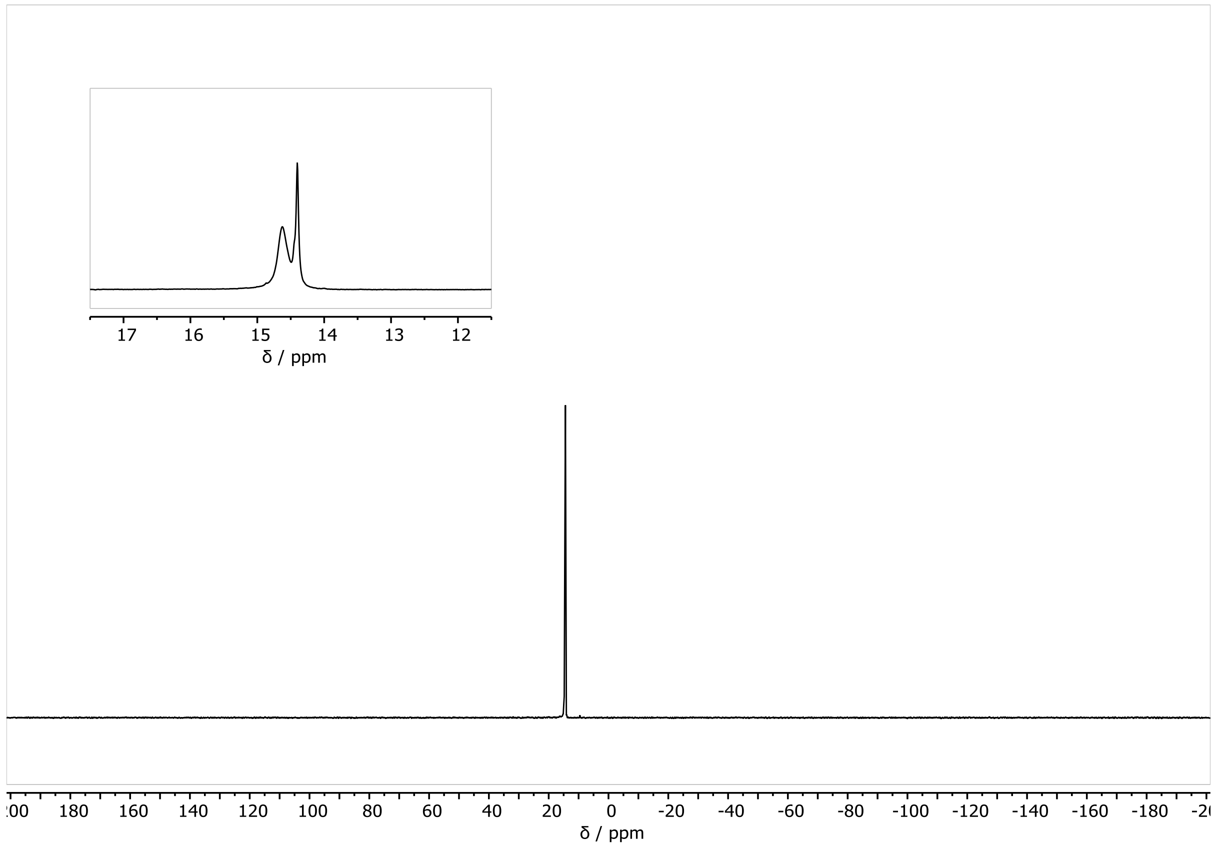

Figure S39. ^{31}P NMR (202 MHz, DMSO-d$_6$) of **DPW-3**

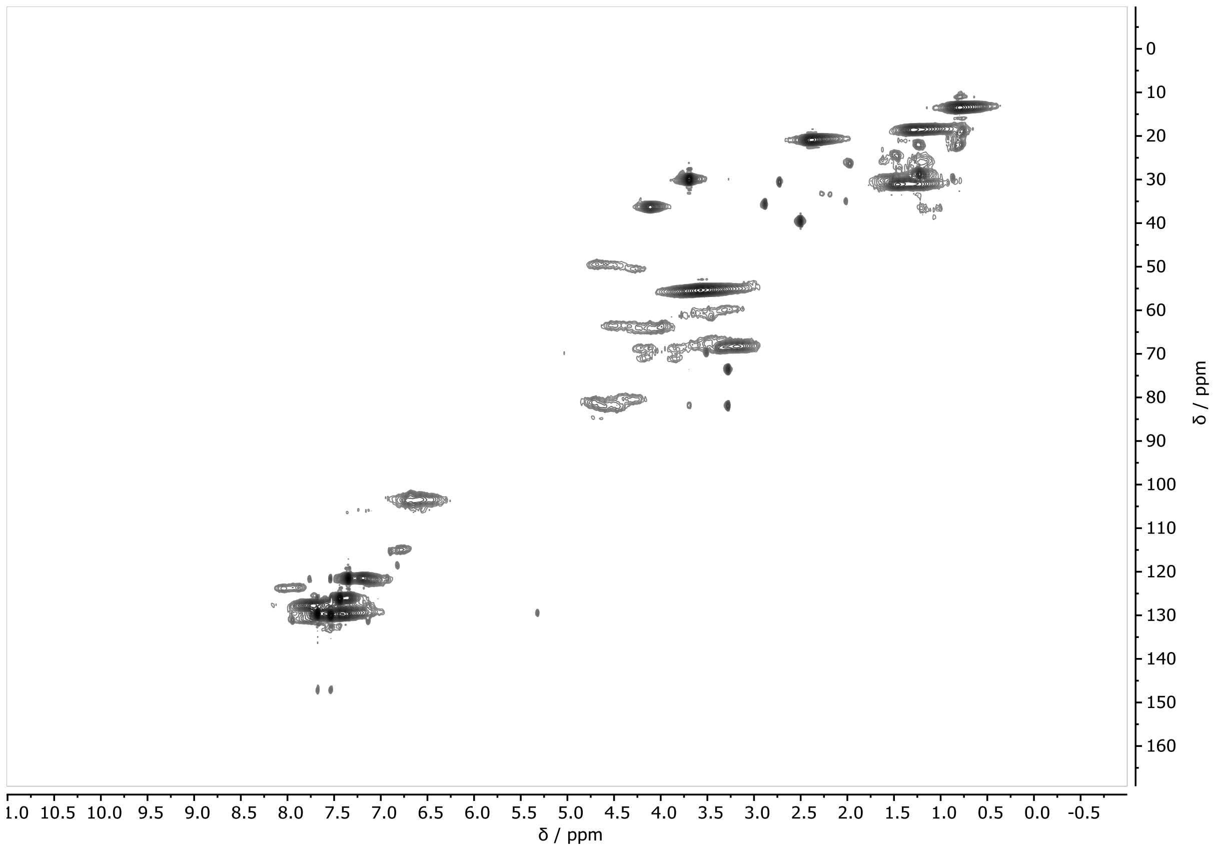

Figure S40. 2D HSQC NMR (700 MHz, DMSO-d$_6$) of **DPW-6**

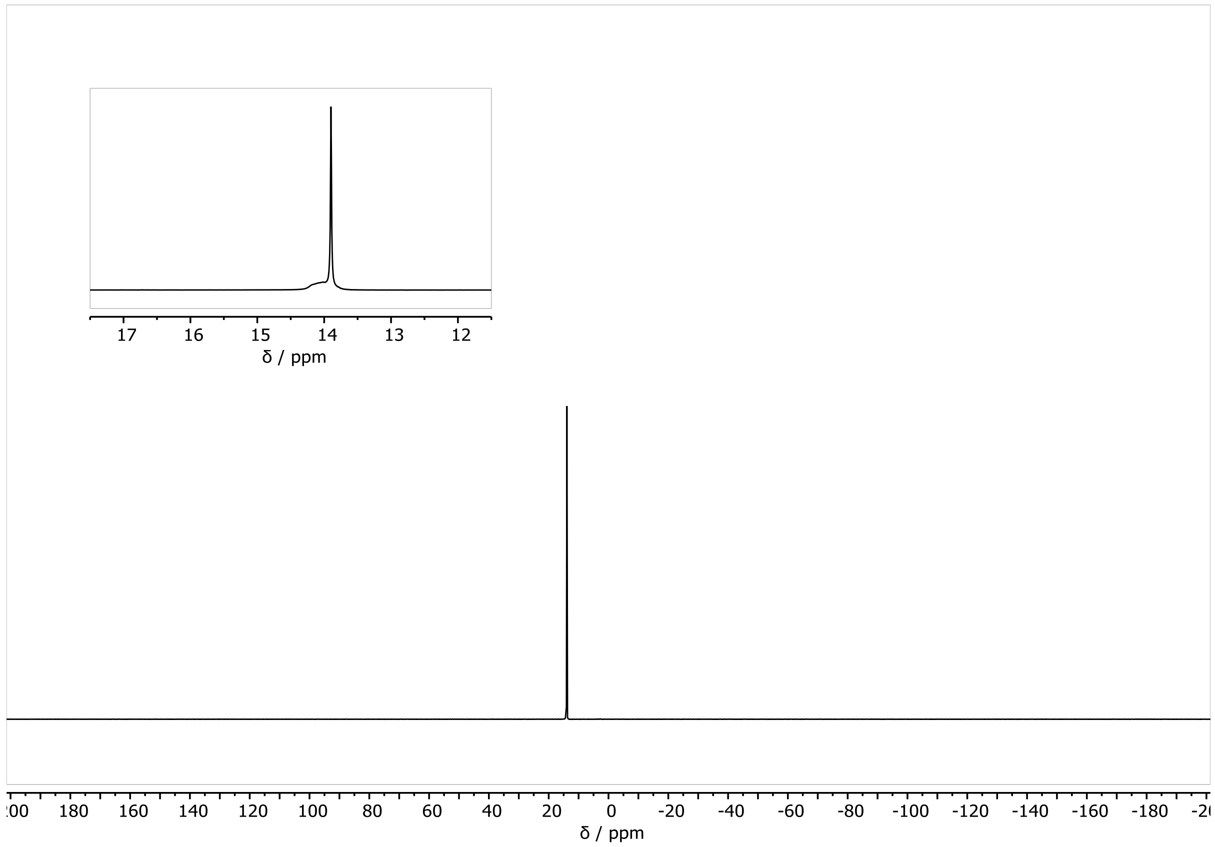

Figure S41. ^{31}P NMR (202 MHz, DMSO-d$_6$) of **DPW-6**

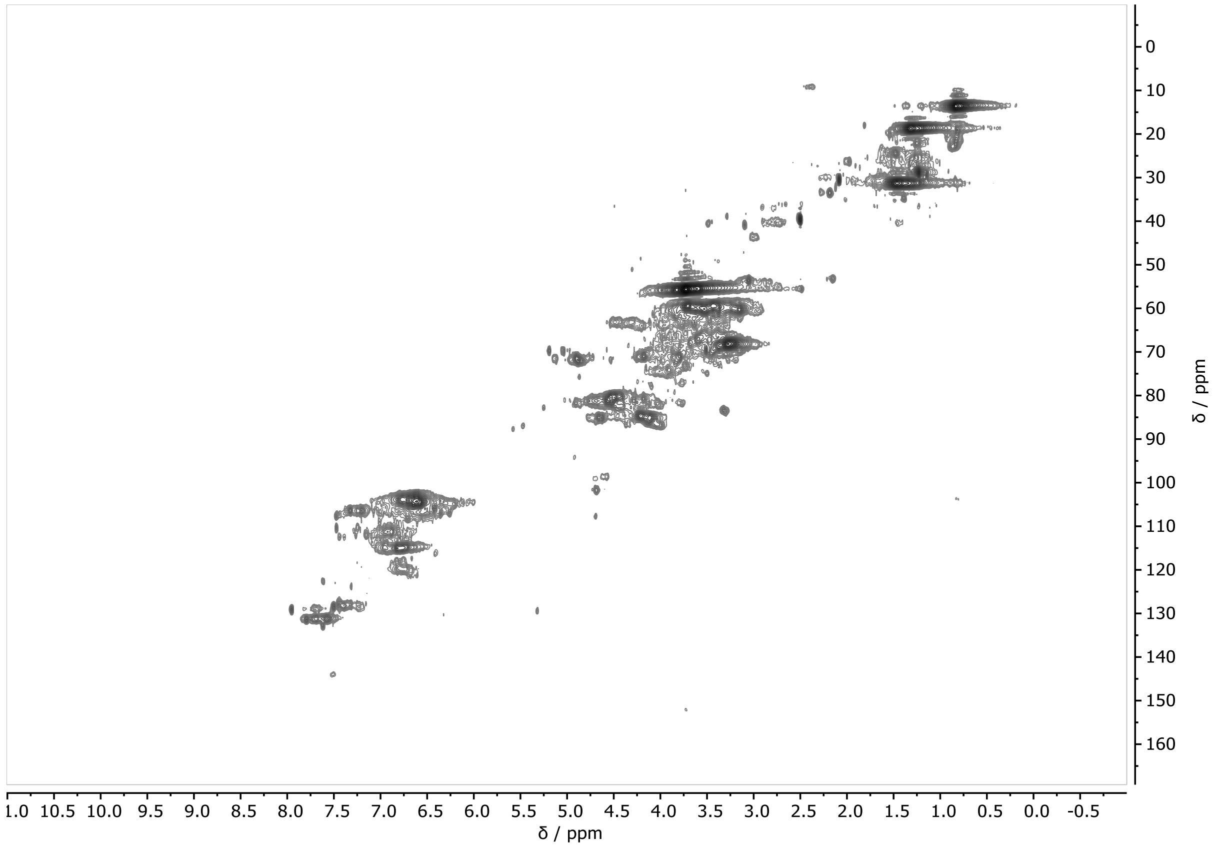

Figure S42. 2D HSQC NMR (700 MHz, DMSO-d$_6$) of **DeAcyl Lignin**

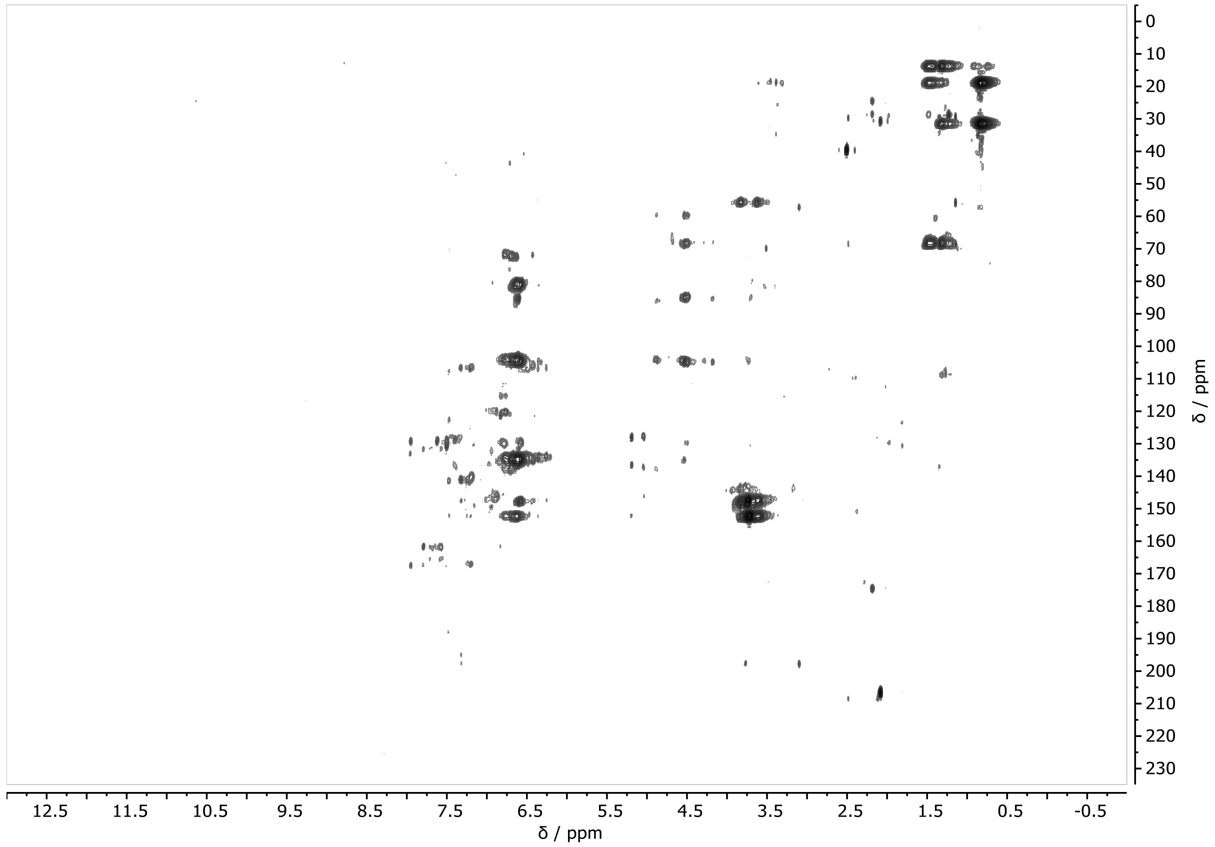

Figure S43. 2D HMBC NMR (700 MHz, DMSO-d$_6$) of **DeAcyl Lignin**

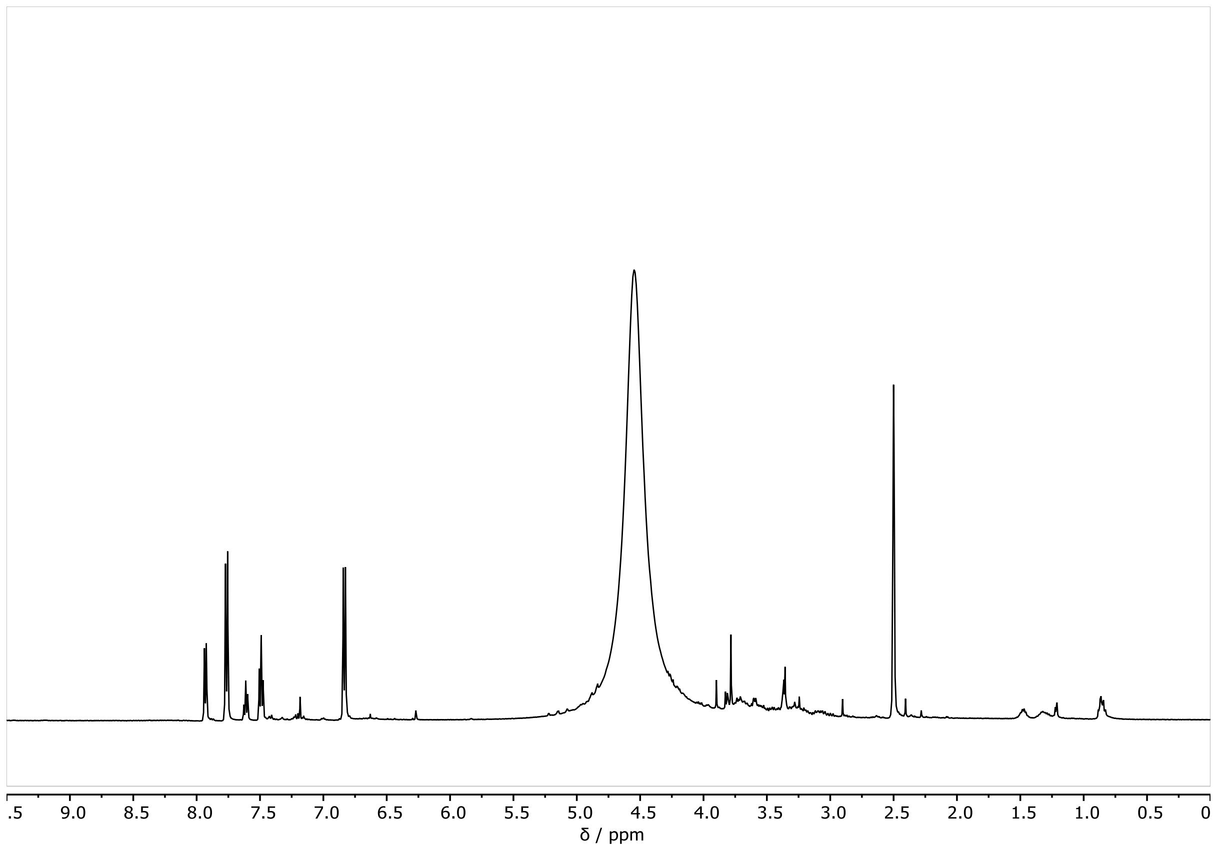

Figure S44. ^{1}H NMR (700 MHz, DMSO-d$_6$) of aqueous component from hydrolysis of **DPW Lignin**

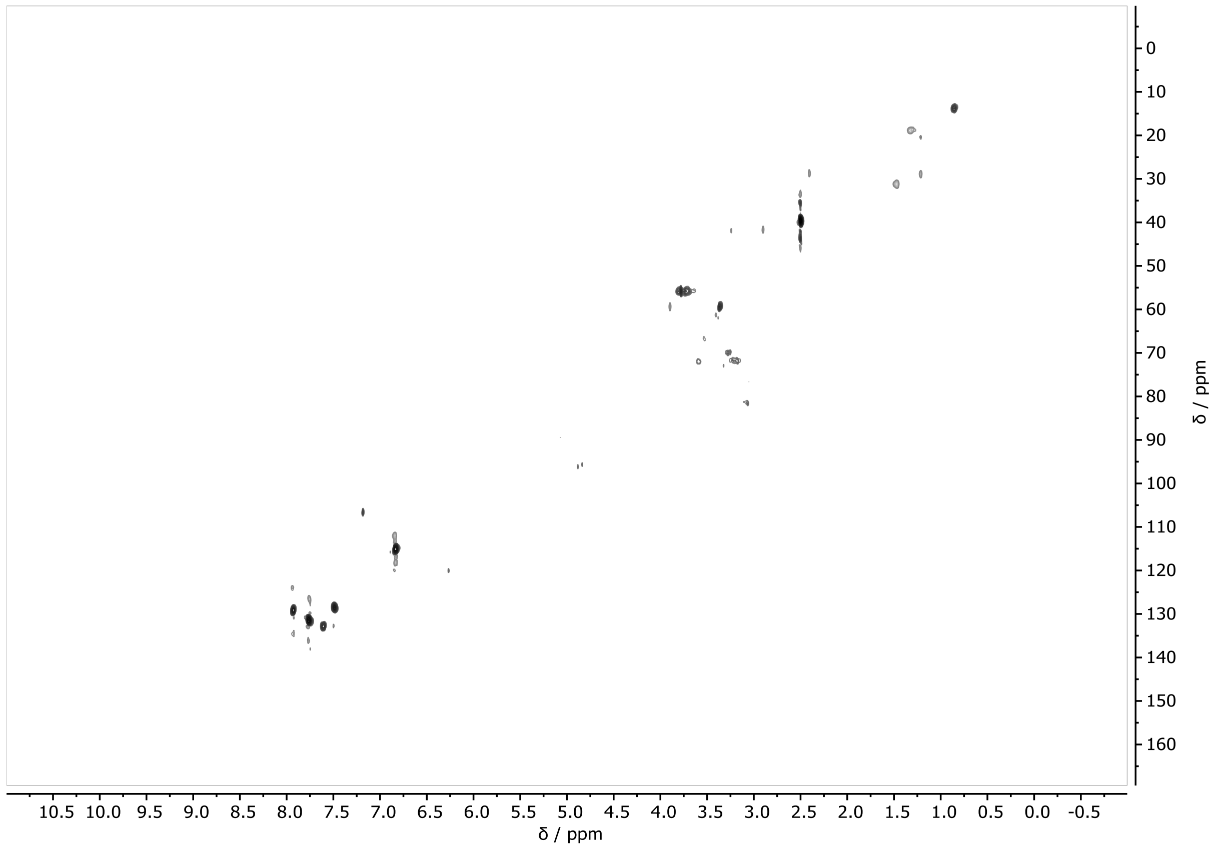

Figure S45. 2D HSQC NMR (700 MHz, DMSO-d$_6$) of aqueous component from hydrolysis of **DPW Lignin**

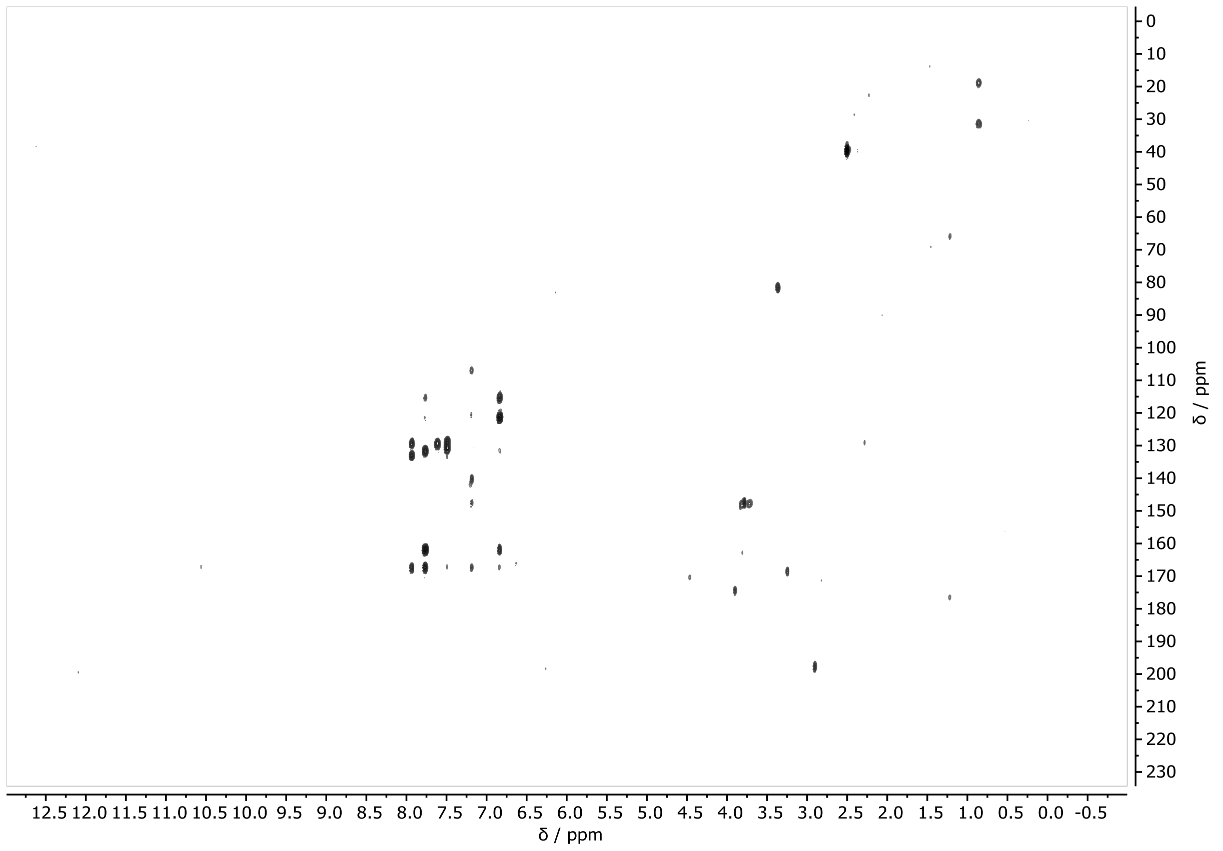

Figure S46. 2D HMBC NMR (700 MHz, DMSO-d$_6$) of aqueous component from hydrolysis of **DPW Lignin**

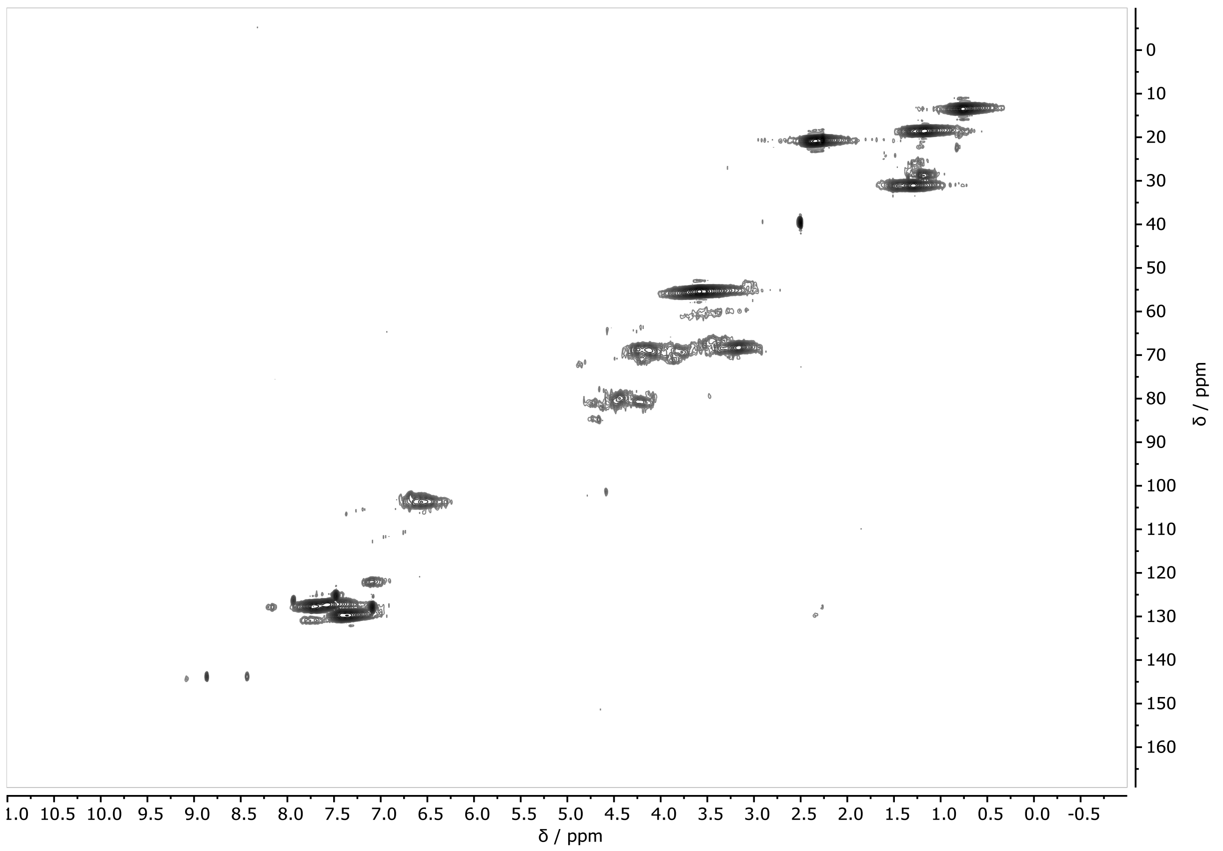

Figure S47. 2D HSQC NMR (700 MHz, DMSO-d$_6$) of **DeAcyl Lignin-Ts**

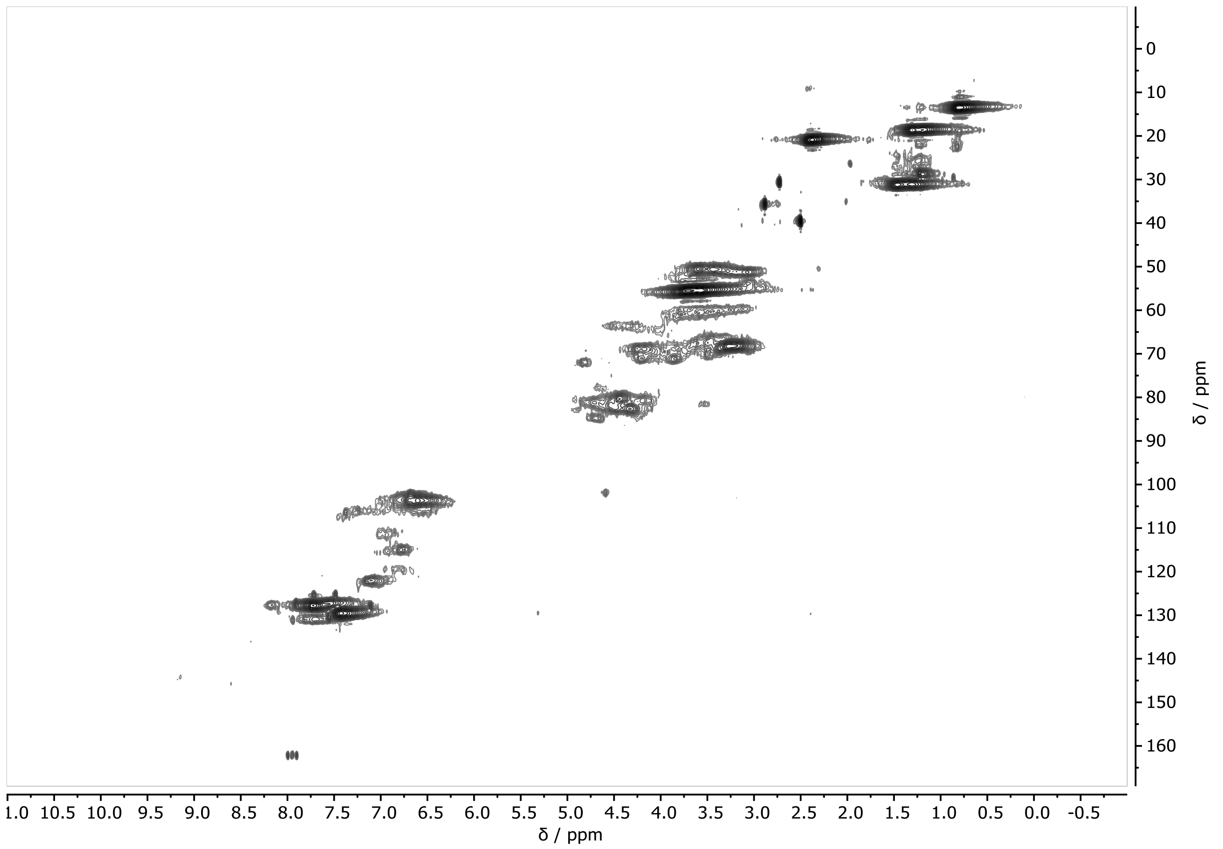

Figure S48. 2D HSQC NMR (700 MHz, DMSO-d_6) of **DeAcyl Lignin-N_3**

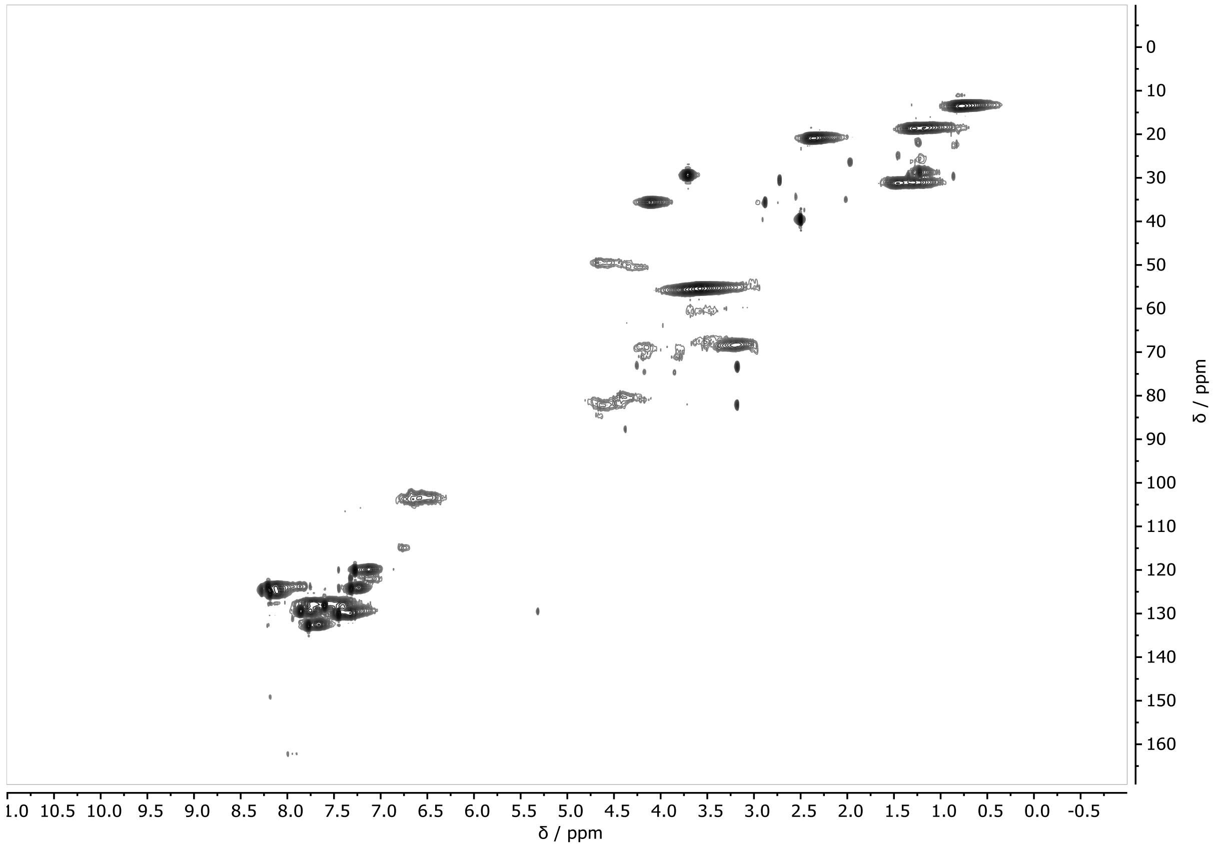

Figure S49. 2D HSQC NMR (700 MHz, DMSO-d_6) of **DeAcyl-3**

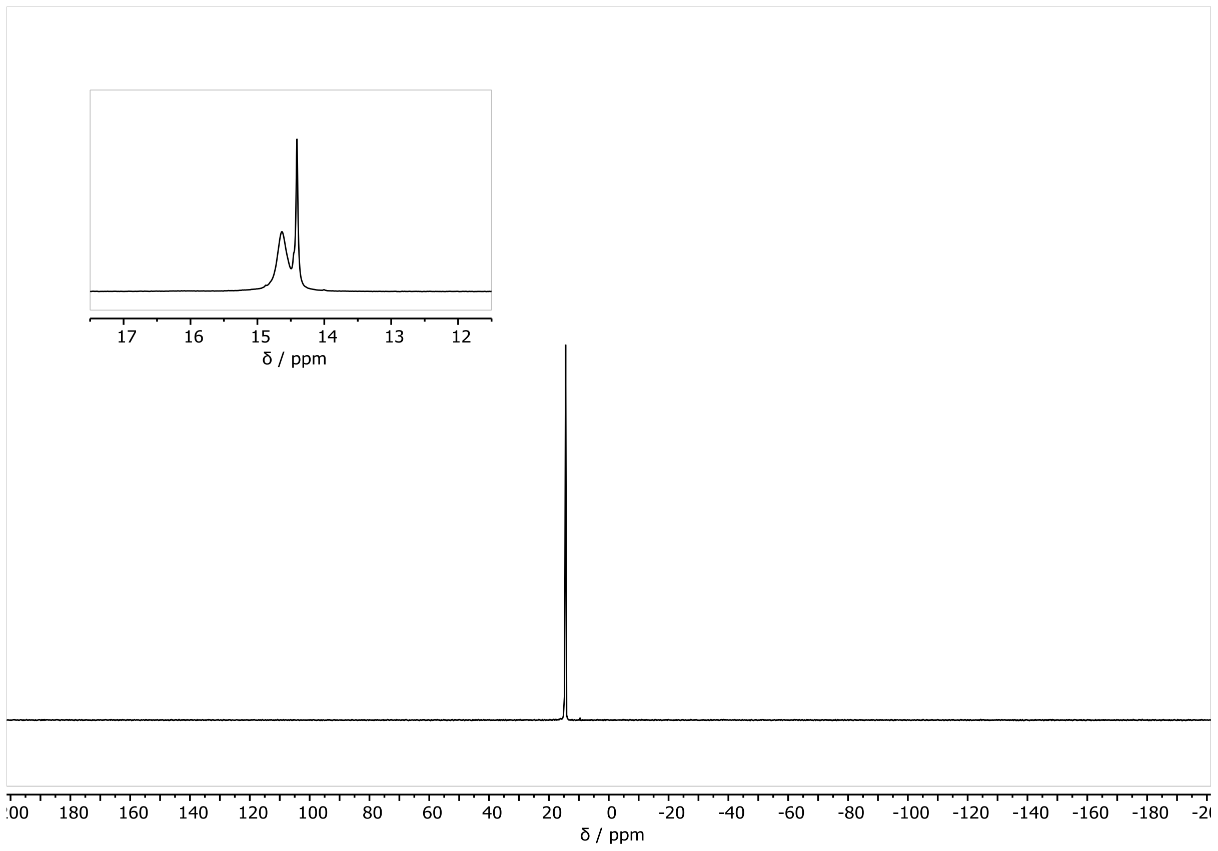

Figure S50. ^{31}P NMR (202 MHz, DMSO-d_6) of **DeAcyl-3**

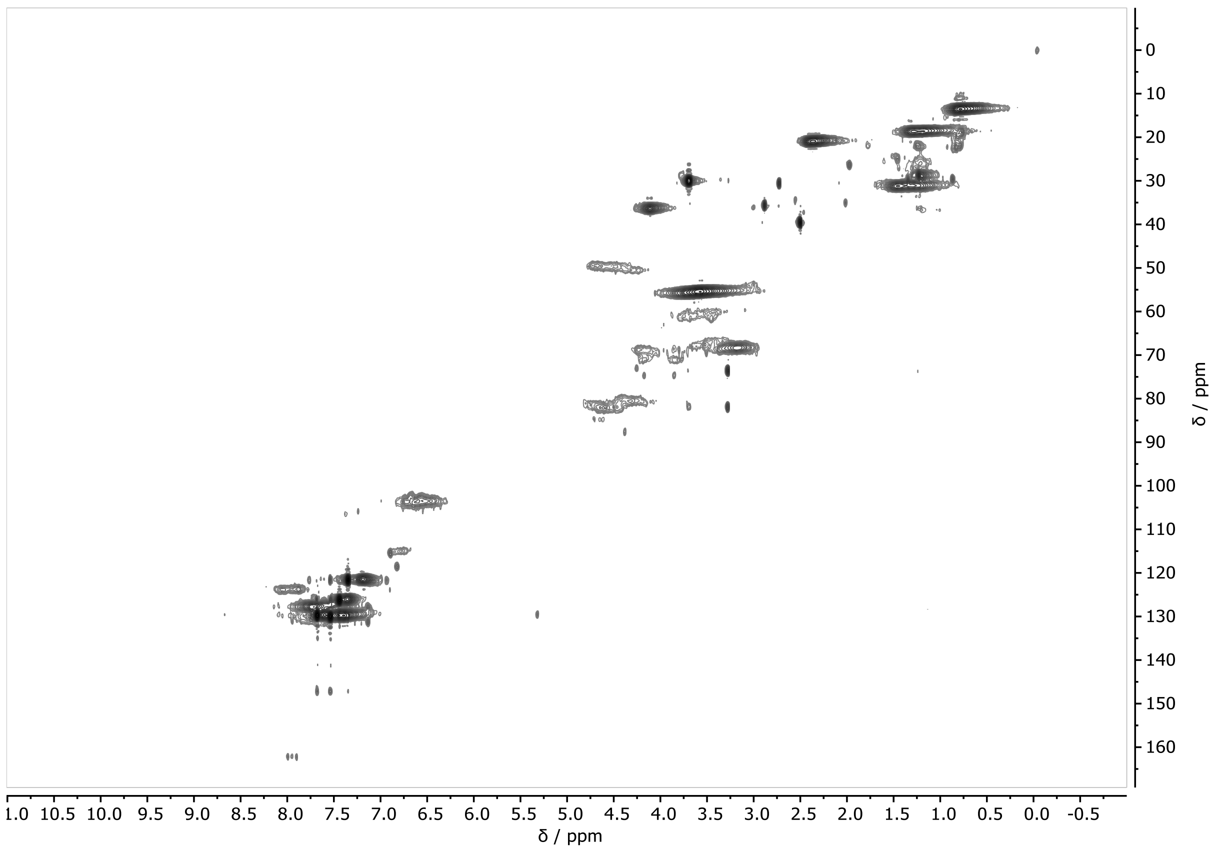

Figure S51. 2D HSQC NMR (700 MHz, DMSO-d_6) of **DeAcyl-6**

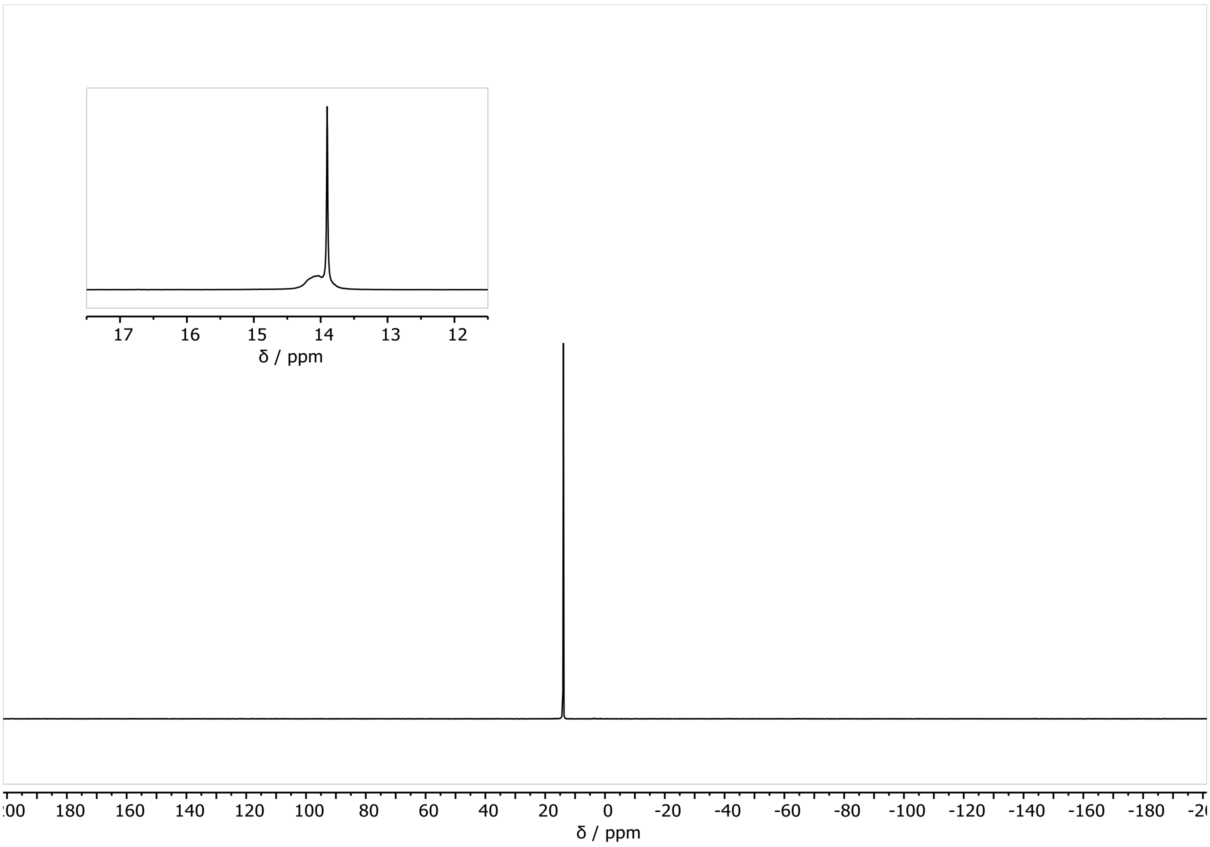

Figure S52. ^{31}P NMR (202 MHz, DMSO-d_6) of **DeAcyl-6**

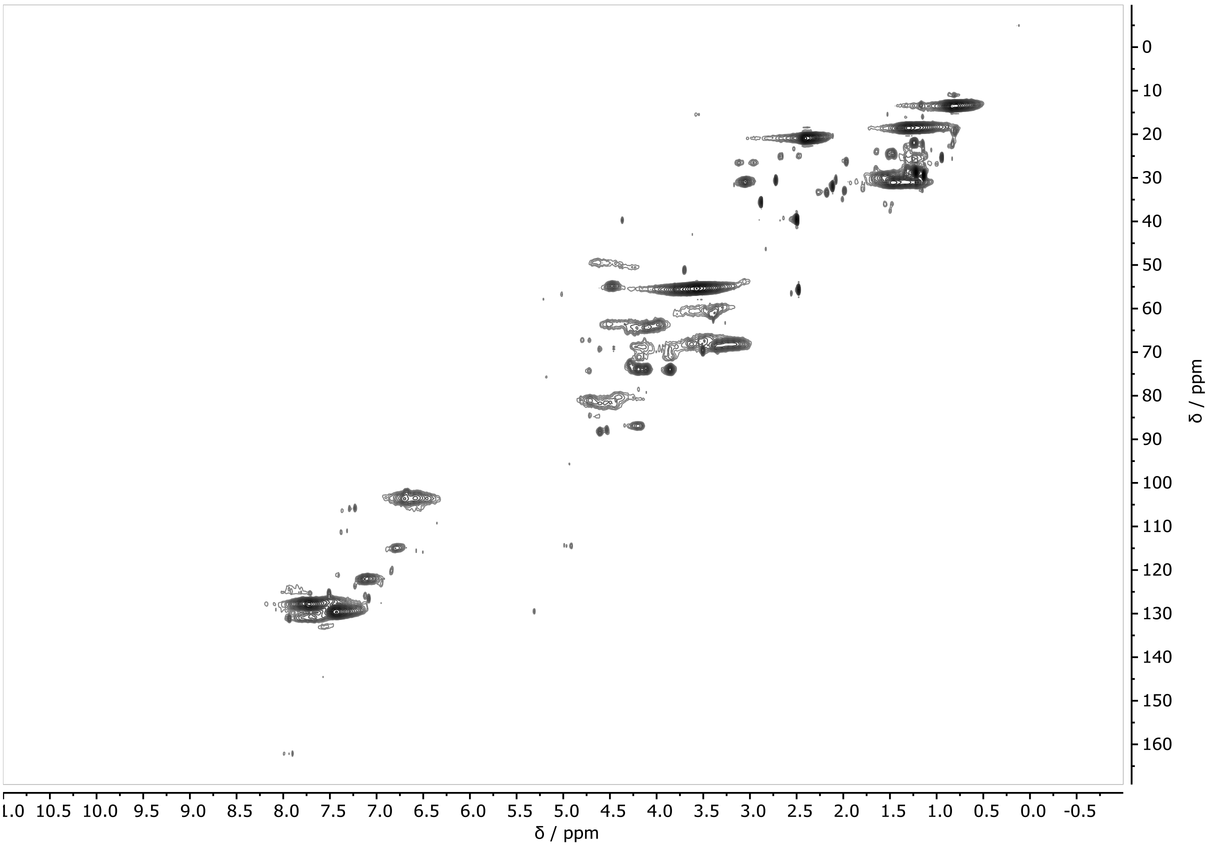

Figure S53. 2D HSQC NMR (700 MHz, DMSO-d_6) of **DPW Lignin** after attempted modification with **5**

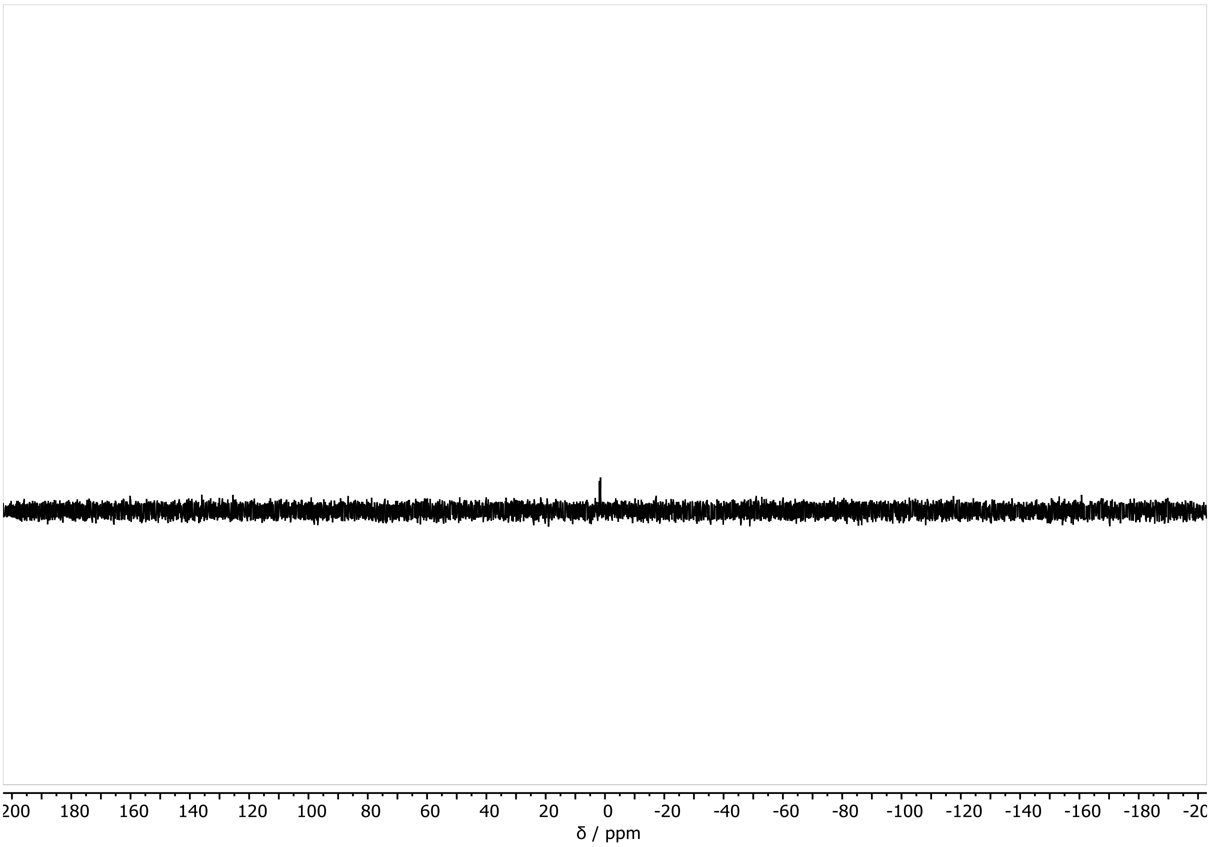

Figure S54. ^{31}P NMR (202 MHz, DMSO-d_6) of **DPW Lignin** after attempted modification with **5**

Supplementary Material References

1. Davidson, D.J.; Lu, F.; Faas, L.; Dawson, D.M.; Warren, G.P.; Panovic, I.; Montgomery, J.R.D.; Ma, X.; Bosilkov, B.G.; Slawin, A.M.Z.; et al. Organosolv Pretreatment of Cocoa Pod Husks: Isolation, Analysis, and Use of Lignin from an Abundant Waste Product. ACS Sustain. Chem. Eng., 2023, 11, 14323–14333, doi:10.1021/ACSSUSCHEMENG.2C03670.
2. Panovic, I.; Montgomery, J.R.D.; Lancefield, C.S.; Puri, D.; Lebl, T.; Westwood, N.J. Grafting of Technical Lignins through Regioselective Triazole Formation on β-O-4 Linkages. ACS Sustain. Chem. Eng., 2017, 5, 10640–10648, doi:10.1021/acssuschemeng.7b02575.
3. Granata, A.; Argyropoulos, D.S. 2-Chloro-4,4,5,5-Tetramethyl-1,3,2-Dioxaphospholane, a Reagent for the Accurate De-termination of the Uncondensed and Condensed Phenolic Moieties in Lignins. J. Agric. Food Chem., 1995, 43, 1538–1544, doi.org/10.1021/jf00054a023.
4. Jiang, Z. -H; Argyropoulos, D.S.; Granata, A. Correlation Analysis of 31P NMR Chemical Shifts with Substituent Effects of Phenols. Magn. Reason. Chem., 1995, 33, 375–382, doi:10.1002/mrc.1260330509.
5. Karlen, S.D.; Smith, R.A.; Kim, H.; Padmakshan, D.; Bartuce, A.; Mobley, J.K.; Free, H.C.A.; Smith, B.G.; Harris, P.J.; Ralph, J. Highly Decorated Lignins in Leaf Tissues of the Canary Island Date Palm Phoenix Canariensis. Plant Physiol., 2017, 175, 1058–1067, doi:10.1104/PP.17.01172.
